# Varenicline Combined With Oral Nicotine Replacement Therapy and Smartphone-Based Medication Reminders for Smoking Cessation: Feasibility Randomized Controlled Trial

**DOI:** 10.2196/48857

**Published:** 2023-10-27

**Authors:** Munjireen Sifat, Emily T Hébert, Jasjit S Ahluwalia, Michael S Businelle, Joseph J C Waring, Summer G Frank-Pearce, Chase Bryer, Lizbeth Benson, Stefani Madison, Lourdes G Planas, Irina Baranskaya, Darla E Kendzor

**Affiliations:** 1 Tobacco Settlement Endowment Trust Health Promotion Research Center Stephenson Cancer Center University of Oklahoma Health Sciences Center Oklahoma City, OK United States; 2 Medical Oncology Sidney Kimmel Cancer Center Thomas Jefferson University Philadelphia, PA United States; 3 School of Public Health The University of Texas Health Science Center Austin, TX United States; 4 Department of Behavioral and Social Sciences Brown University School of Public Health Providence, RI United States; 5 Department of Family and Preventive Medicine University of Oklahoma Health Sciences Center Oklahoma City, OK United States; 6 Department of Mental Health Johns Hopkins Bloomberg School of Public Health Johns Hopkins University Baltimore, MD United States; 7 Department of Biostatistics and Epidemiology Hudson College of Public Health University of Oklahoma Health Sciences Center Oklahoma City, OK United States; 8 Department of Pharmacy: Clinical and Administrative Sciences University of Oklahoma Health Sciences Center Oklahoma City, OK United States; 9 Department of Psychiatry and Behavioral Sciences University of Oklahoma Health Sciences Center Oklahoma City, OK United States

**Keywords:** mHealth, smartphone-based medication reminders, varenicline combined with oral nicotine replacement therapy, smoking cessation, medication adherence, smoking, smoker, smoke, cessation, quit, quitting, nicotine, nicotine replacement therapy, NRT, randomized, controlled trial, mobile phone

## Abstract

**Background:**

Varenicline and oral nicotine replacement therapy (NRT) have each been shown to increase the likelihood of smoking cessation, but their combination has not been studied. In addition, smoking cessation medication adherence is often poor, thus, challenging the ability to evaluate medication efficacy.

**Objective:**

This study examined the effects of combined varenicline and oral NRT and smartphone medication reminders on pharmacotherapy adherence and smoking abstinence among adults enrolled in smoking cessation treatment.

**Methods:**

A 2×2 factorial design was used. Participants (N=34) were randomized to (1) varenicline + oral NRT (VAR+NRT) or varenicline alone (VAR) and (2) smartphone medication reminder messages (REM) or no reminder messages (NREM) over 13 weeks. Participants assigned to VAR+REM received varenicline reminder prompts, and those assigned to VAR+NRT+REM also received reminders to use oral NRT. The other 2 groups (VAR+NREM and VAR+NRT+NREM) did not receive medication reminders. Participants were not blinded to intervention groups. All participants received tobacco cessation counseling. Smartphone assessments of smoking as well as varenicline and NRT use (if applicable) were prompted daily through the first 12 weeks after a scheduled quit date. Descriptive statistics were generated to characterize the relations between medication and reminder group assignments with daily smoking, daily varenicline adherence, and daily quantity of oral NRT used. Participants completed follow-up assessments for 26 weeks after the quit date.

**Results:**

Participants were predominantly White (71%), and half were female (50%). On average, participants were 54.2 (SD 9.4) years of age, they smoked an average of 19.0 (SD 9.0) cigarettes per day and had smoked for 34.6 (SD 12.7) years. Descriptively, participants assigned to VAR+NRT reported more days of smoking abstinence compared to VAR (29.3 vs 26.3 days). Participants assigned to REM reported more days of smoking abstinence than those assigned to NREM (40.5 vs 21.8 days). Participants assigned to REM were adherent to varenicline on more days compared to those assigned to NREM (58.6 vs 40.5 days), and participants assigned to VAR were adherent to varenicline on more days than those assigned to VAR + NRT (50.7 vs 43.3 days). In the subsample of participants assigned to VAR+NRT, participants assigned to REM reported more days where ≥5 pieces of NRT were used than NREM (14.0 vs 7.4 days). Average overall medication adherence (assessed via the Medication Adherence Questionnaire) showed the same pattern as the daily smartphone-based adherence assessments.

**Conclusions:**

Preliminary findings indicated that smoking cessation interventions may benefit from incorporating medication reminders and combining varenicline with oral NRT, though combining medications may be associated with poorer adherence. Further study is warranted.

**Trial Registration:**

ClinicalTrials.gov NCT03722966; https://classic.clinicaltrials.gov/ct2/show/NCT03722966

## Introduction

Smoking is the leading modifiable cause of cancer and cancer mortality in the United States [1]. Although the prevalence of smoking has declined over the past 5 decades, approximately 12.5% of adults continue to smoke in the United States [2]. Smoking is known to cause nearly 20% of all cancers, 30% of cancer deaths [1], and approximately 80% of lung cancers [3]. Smoking cessation increases life expectancy, and quitting at an earlier age is associated with more years of life gained than quitting later in life [4]. Effective pharmacotherapies to aid cessation are available, and the combination of behavioral support with pharmacotherapy is associated with optimal cessation outcomes [5]. More recently, research has focused on evaluating combination pharmacotherapies in an effort to refine current evidence-based treatment approaches.

Varenicline is considered a first-line tobacco cessation treatment [6]. Varenicline and nicotine replacement therapy (NRT) each promote smoking cessation via potentially complementary pathways. Varenicline is a partial nicotine receptor agonist and also has nicotine receptor antagonist properties. The agonist effect of varenicline is about half the effect of nicotine at the receptor, thereby reducing craving and withdrawal during periods of smoking abstinence while also competing with nicotine for binding at the receptor site, thereby making smoking less reinforcing [7,8]. The nicotine in lozenges is a full nicotine receptor agonist, thus, reducing craving and withdrawal similar to nicotine in cigarettes [9]. However, the slower absorption of nicotine in lozenges through the buccal mucosa (vs the lungs with cigarette smoking) along with the absence of other additive and addictive compounds (eg, acetaldehyde and menthol) reduces the addiction potential of oral NRT [9]. Since varenicline is a partial agonist and may not fully saturate nicotine receptors, NRT can supplement the effects of varenicline both preemptively and during acute cravings [10].

The combination of varenicline and nicotine patches has been evaluated in several randomized trials [10-14]. Two meta-analyses have indicated that varenicline combined with the nicotine patch was more effective than varenicline alone for promoting smoking abstinence at 3- and 6-month follow-up across studies [15,16]. However, no published studies to date have reported on the combined impact of varenicline and oral NRT on smoking cessation in a full-scale randomized trial. Oral NRT provides acute relief from cravings [17] and offers individuals the flexibility to deliver nicotine when they need it most in contrast with the continuous and passive nicotine delivery of nicotine via the patch. With oral NRT, peak plasma nicotine levels are reached within 20-30 minutes and typically return to baseline within about 2 hours (ie, active, rapid, and short-acting) [17]. In contrast, peak nicotine levels are reached after about an hour with the nicotine patch and last for about 24 hours (ie, passive, delayed, and long-acting) [17]. Thus, oral NRT may be particularly useful as a supplement to other effective treatments, such as varenicline, for acute cravings. Nevertheless, in clinical trials of other medication combinations, such as bupropion and nicotine gum, participants’ use of nicotine gum was infrequent [18,19], which can make it difficult to determine whether there is an added benefit of combined oral NRT with other medication.

Given the near ubiquity of smartphone ownership (ie, 85% of US adults) [20], smartphone-based medication adherence interventions offer a means to improve smoking cessation pharmacotherapy adherence [21,22]. Specifically, smartphone medication reminders are a promising strategy to improve adherence to treatments [23]. Medication adherence is important, particularly in the early weeks of a smoking cessation attempt, because better medication adherence is associated with a greater likelihood of achieving smoking cessation [24-26]. For example, previous research has shown that a greater blood level of bupropion early in treatment was associated with long-term biochemically verified smoking abstinence [27]. Key preventable causes of medication nonadherence include forgetfulness and beliefs that smoking cessation medications are not needed or do not help with cessation [28]. Smartphone apps that provide medication reminders in real time have been shown to increase health knowledge and support for medication adherence [21]. Reviews of interventions for certain conditions, including cardiovascular disease and hypertension, have demonstrated positive impacts of app-based reminders on medication adherence [29-31]. Only a couple of medication management apps have specifically focused on increasing smoking cessation medication adherence with initial evidence of feasibility [21,22]. Further study is needed to evaluate the effectiveness of smartphone-based smoking cessation medication reminders, given the initial evidence of efficacy, high scalability, and relatively low cost [32].

The purpose of this pilot study was to characterize the feasibility and preliminary efficacy of a combination of varenicline and oral NRT among adults initiating smoking cessation treatment. In addition, this study described the potential impact of smartphone-based medication reminder prompts on medication adherence and smoking cessation. Findings will provide initial information regarding the possible benefits of a combination of varenicline and oral NRT and smartphone medication reminders and inform decisions about whether further investigation is warranted.

## Methods

### Participants

Participants were recruited for the study via tobacco treatment referrals to the Tobacco Treatment Research Program (TTRP) in Oklahoma City [33] and through social media advertising in the surrounding areas. In addition, adults who had previously completed standard treatment at the TTRP but had not quit smoking after 6 months were recruited. Individuals were eligible for the study if they (1) were ≥18 years of age, (2) demonstrated higher than sixth-grade English literacy level (assessed via the Short-Form Rapid Estimate of Adult Literacy in Medicine [34]), (3) agreed to install the study smartphone app onto their personal phone or were willing to carry a study-provided phone with the app, (4) had an expired carbon monoxide (CO) level >6 ppm suggestive of current smoking [35], (5) reported smoking ≥5 cigarettes per day at the time of enrollment, (6) were willing to initiate a quit attempt ≈7 days after enrollment, (7) were willing to use nicotine gum or lozenges, and (8) were eligible to use varenicline after consultation with the study physician. Individuals were excluded from the study if they (1) had a history of seizures, (2) were allergic to varenicline, (3) were considered to be at high risk for suicide (score ≥10 on the Mini-International Neuropsychiatric Interview Suicidal Scale [36]), or (4) were pregnant, planning to become pregnant, or currently breastfeeding at the time of enrollment.

### Procedure

#### Overview

This study used a 2×2 factorial design (Table 1). Participants (N=34) were randomized to (1) varenicline + nicotine gum or lozenges (VAR+NRT; n=20) or varenicline alone (VAR; n=14) and (2) smartphone medication reminder messages (REM; n=11) or no reminder messages (NREM; n=23). Adaptive randomization [37,38] was used to assign participants to groups based on race, sex, cigarettes smoked per day, and education. Participants assigned to VAR+REM (n=2) received varenicline reminder prompts, and those assigned to VAR+NRT+REM (n=9) also received reminders to use gum or lozenges with decreasing frequency over time. Participants assigned to VAR+NREM (n=12) and VAR+NRT+NREM (n=11) did not receive medication reminders. Participants were followed for 27 weeks, beginning 1 week before their scheduled quit date through 26 weeks after their quit date, with key follow-up assessments of smoking status at 4, 8, 12, and 26 weeks post quit. Participants also completed daily smartphone assessments from 1 week before their quit date through 12 weeks after their quit date. All participants were enrolled between January 2020 and July 2021, and final 26-week follow-up visits were completed by February 2022. The study was discontinued early due to the Pfizer recall of varenicline (Chantix) following findings that nitrosamine levels were above the US Food and Drug Administration [39] acceptable intake limit. However, note that generic varenicline has become available since the conclusion of the study and can be accessed in future research.

**Table 1:** Factorial design (2×2).

		Medication type	
		VAR^a^	VAR + oral NRT^b^ (VAR + NRT)
**Reminders**			
	REM^c^	VAR + REM (n=2)	VAR + NRT + REM (n=9)
	NREM^d^	VAR + NREM (n=12)	VAR + NRT + NREM (n=11)

^a^VAR: varenicline.

^b^NRT: nicotine replacement therapy.

^c^REM: reminder.

^d^NREM: no reminder.

#### Assessment Schedule and Compensation

Participants were asked to complete web-based assessments via the Research Electric Data Capture (REDCap; Vanderbilt University [40,41]) program at baseline (1 week before quitting) and then weekly through 4 weeks post quit with additional follow-up assessments at 8, 12, and 26 weeks post quit. Participants were compensated US $20 for each completed assessment (up to US $120).

Participants were prompted via the Insight smartphone app [42] to complete daily diary assessments 30 minutes after their self-reported usual wake time. Daily smoking, medication adherence, and other relevant variables were assessed from 1 week prior to the scheduled quit date through 12 weeks post quit. Participants also received medication reminder prompts (described in the Medication REM section). Those who responded that they did not take their medication as prescribed were asked about their reasons for nonadherence. Participants were compensated up to US $10 per week for the completion of daily diary assessments (up to US $130 total). Specifically, participants earned US $10 for completing 6-7 daily diary assessments or US $5 for completing 4-5 assessments each week. Participants who completed ≤3 daily diary assessments did not receive compensation for that week. The primary study outcomes were CO-confirmed, self-reported 7-day point prevalence abstinence at 4, 8, 12, and 26 weeks postquit follow-up.

Participants were initially asked to complete study follow-up assessments in person at the TTRP, but the onset of the COVID-19 pandemic led the university to limit in-person interactions on campus. As a result, between March and May 2020, all assessments were completed remotely via web-based assessments, daily smartphone assessments, and smartphone-based breath sample submissions. After the university reopened, participants were encouraged to attend study visits in person, but remote assessments were accommodated.

#### Counseling

Approximately 1-week prior to the scheduled quit date, a Tobacco Treatment Specialist (TTS) provided an overview of the tobacco cessation program and assisted participants with developing a quit plan. Participants were instructed to quit smoking at bedtime or 10 PM (whichever occurred first) on the evening before their next weekly counseling session (≈1 week after enrollment). All participants were offered up to 5 additional weekly counseling sessions delivered in-person or by telephone with a TTS. Topics of discussion during the counseling sessions included (1) the impact of tobacco on the health benefits of quitting, (2) stress management strategies, (3) making positive lifestyle changes, (4) developing coping skills, and (5) relapse prevention. The TTS checked in with participants each week about the difficulties and successes they experienced and planned for anticipated future challenges.

#### Varenicline

Participants were evaluated by a study physician, and varenicline was prescribed. Doses were titrated during the first week (prequit) following enrollment (days 1-3: 0.5 mg once daily; days 4-7: 0.5 mg twice daily). Thereafter, participants were instructed to take 0.5 mg varenicline twice daily for 12 additional postquit weeks.

#### Oral NRT

Participants randomly assigned to VAR + NRT were provided a supply of 2 or 4 mg nicotine lozenges or gum based on their prequit level of smoking (per package instructions) and their preference for gum or lozenges. Participants were instructed to start using gum or lozenges on their quit date and especially when they had the urge to smoke. Participants were initially instructed to use gum or lozenge every 1-2 hours during weeks 1-6 and to gradually reduce to every 2-4 hours during weeks 7-9 and 4-8 hours during weeks 10-12 after the scheduled quit date (per package instructions).

#### Medication REM

Participants randomly assigned to receive varenicline reminders received these reminders at times selected based on their preference (morning and evening after full titration), for example, “Don’t forget to take your Chantix pill this morning!” The reminders also inquired about whether the participant had taken their medication, for example, “You were scheduled to take your morning Chantix pill at 9 AM. Have you taken your Chantix pill this morning?” Participants were sent reminder messages twice before being asked about the main reason they did not take missed varenicline dose. Reminder prompts for varenicline began during the prequit period of the study.

Participants assigned to receive NRT reminders also received reminders to use NRT throughout the day, such as “Don't forget to use nicotine gum/lozenges regularly throughout the day! It is recommended that you use a piece of gum/lozenge every 1-2 hours.” They received up to 3 additional prompts to report whether or not they used a piece of gum or a lozenge. Participants received reminders every 3 hours from the scheduled quit day to 4 weeks post quit, every 4 hours during weeks 5-8, and every 6 hours during weeks 9-12 after the scheduled quit date. Note that reminder messages were delivered less frequently than the recommended dose of NRT (eg, every 1-2 hours during the first 6 weeks) due to concerns that the high frequency of reminder messages might be perceived as aversive or burdensome.

### Ethics Approval

This study was approved by the institutional review board of the University of Oklahoma Health Sciences Center (protocol 10184). Informed consent was obtained from all participants. Privacy and confidentiality were maintained by assigning an identification number in place of names in all secured electronic and print data files. Participants were able to earn up to US $250 in total compensation (gift cards) for assessment completions over the entire study period.

### Measures

#### Sociodemographic Characteristics

Participants self-reported their sex (male vs female), race (White vs racially minoritized [Black or African American, American Indian or Alaska Native, Asian or Pacific Islander, and multirace or other]), ethnicity (Hispanic vs non-Hispanic), years of age, education level (under high school vs under or above high school), and annual household income (<US $50,000 vs ≥US $50,000).

#### Tobacco History

Participants reported the average number of cigarettes smoked per day prior to quitting and the number of years they had smoked at the time of enrollment.

#### Smoking Abstinence

The primary study outcomes were self-reported and biochemically verified 7-day point prevalence abstinence at 4, 8, 12, and 26 weeks after the scheduled quit date. CO was assessed using a portable Vitalograph ecolyzer (Vitalograph Inc; for in-clinic assessments) or via the Bedfont iCO Smokerlyzer (Bedfont Scientific Ltd; for remote smartphone-based assessments) with a CO level of ≤6 ppm indicating biochemical confirmation of self-reported abstinence (per current recommendations [35]).

Participants were considered abstinent each day if they did not report smoking during any smartphone-based reminder assessment on that day, and they reported that they did not smoke during the previous day on the morning daily diary. A variable reflecting the total number of days each participant was abstinent over the postquit period was created with a possible range of 0-84 days abstinent (ie, the first 12 weeks after the scheduled quit date).

#### Varenicline Adherence

Participants responded to the following smartphone daily diary question each morning and evening (depending on where they were in the titration schedule): “Have you taken your Chantix pill this [morning/evening]?” A dichotomous variable was created to indicate whether or not the participant had reported using varenicline as prescribed on a given day. For example, if a participant was supposed to take the medication once in the morning and once in the evening, but they reported only taking it once, they were considered nonadherent for that day. The total days of varenicline adherence were calculated with a possible range of 0-91 days (ie, 1 week before the quit date [titration period] through 12 weeks after the scheduled quit date).

#### NRT Adherence

During each morning daily diary, participants were asked via smartphone assessment, “How many pieces of nicotine gum or lozenges did you use yesterday?” The total pieces of NRT used were reflected as a continuous variable and were later dichotomized to <5 versus ≥5 pieces for each day.

The Medication Adherence Questionnaire (MAQ) [43] is a 4-item participant self-report of adherence to their smoking cessation medications during the previous week. Scores may range from 0 to 4 with higher scores indicating greater medication adherence. The MAQ was administered weekly from the scheduled quit date through 4 weeks after the scheduled quit date and again at the 8- and 12-week postquit follow-ups (7 assessments). Weekly scores were averaged across assessments to create an average adherence score. MAQ scores of 0 were assigned to those who informed study staff that they were dropping out of the study or discontinuing study medication during the weeks after they dropped out. Otherwise, a mean substitution for that week was used in place of missing MAQ scores. MAQ scores were dichotomized based on a median split to reflect higher versus lower adherence within the sample (<3.60 vs ≥3.60).

#### Reasons for Nonadherence

During smartphone assessments when participants reported that they had not taken their varenicline or NRT, they were prompted to select the main reason for missing their medication. Participants could choose from the following 7 responses: “I forgot to take it, I was away from home, I experienced side effects, I didn't feel like I needed it, I didn't think it is working, I have decided not to quit smoking,” or “other,” where they were able to write in their response. Similarly, participants who reported missing their NRT could choose from the following 9 options, “I didn't feel like I needed it, I forgot to take it, I didn’t have it with me, I don't think it is working, I ran out, I experienced side effects, I have decided not to quit smoking, I did not like the taste,” and “other,” which included a write-in option.

### Statistical Analyses

Participants’ sociodemographic characteristics were summarized using frequencies with associated percentages (categorical or binary outcomes) or means with SDs (continuous outcomes). Likewise, the percentage of participants who achieved 7-day self-reported and biochemically verified abstinence at follow-up was described by medication type (VAR vs VAR+NRT) and medication reminder group (REM vs NREM). Participants who did not have complete smoking status data at follow-up were considered smoking (ie, self-reported abstinence but did not provide CO breath sample or were missing both self-reported smoking status and CO breath sample). Due to the early discontinuation of the study as a result of the varenicline recall, randomization across groups was uneven, thus, limiting the ability to describe study outcomes across all 4 groups (eg, only 2 participants were randomized to VAR+REM). Thus, descriptive analyses focus on comparisons between the 2 intervention factors (VAR vs VAR+NRT and REM vs NREM).

Participants reported on their tobacco use and medication adherence via smartphone-based daily dairies over the 13-week treatment period (1 week prequit through 12 weeks post quit) with a total of 84 (tobacco use and NRT use) to 91 (varenicline adherence) assessments possible for each outcome. Due to sample size limitations, differences by the treatment factors were described but not compared for statistical significance. Data were aggregated for each individual participant. Daily abstinence and medication adherence were described by medication type (VAR vs VAR+NRT) and reminder group (REM vs NREM).

## Results

### Participant Characteristics

A total of 35 participants initiated enrollment and were randomized to the study group. However, 1 individual dropped out during the baseline visit and was therefore excluded from all study analyses. Participant characteristics are listed in Table 2.

**Table 2:** Participant characteristics (N=34).

Characteristic	Value
Female, n (%)	17 (50)
Racially minoritized, n (%)^a^	10 (29)
Ethnicity (Hispanic), n (%)	2 (6)
Age (years), mean (SD)	54.2 (9.4)
Education (under high school), n (%)	2 (6)
Annual household income <US $50,000, n (%)	17 (50)
Cigarettes smoked per day, mean (SD)	19.0 (9.0)
Years of smoking, mean (SD)	34.6 (12.7)
Carbon monoxide (baseline; ppm), mean (SD)	24.1 (13.1)

^a^Participants were 71.4% (n=25) White, 14.3% (n=5) Black, 2.9% (n=1) American Indian or Alaska Native, 2.9% (n=1) Asian or Pacific Islander, and 8.6% (n=3) multirace.

The median number of counseling sessions completed was 6 (out of 6 possible including the prequit session) with 85% (n=29) of participants completing either 5 or 6 sessions. Overall, participants completed an average of 81% of all prompted smartphone assessments. Participants were adherent to varenicline in 46.35 (SD 32.29; 73%) days. In the subsample of participants assigned to NRT, participants used an average of 4.06 (SD 5.00) pieces of NRT per day, and they had 10.72 (SD 13.06) days where they used ≥ 5 pieces of NRT per day.

The mean MAQ score averaged across all 7 assessments was 3.20 (SD 1.05), and the median was 3.59 (IQR 3.02-3.90; range 0-4). The distribution of averaged MAQ scores was as follows: 0-0.99=5.9% (n=2), 1-1.99=5.9% (n=2), 2-2.99=8.8% (n=3), 3-3.99=55.9% (n=19), and 4=23.5% (n=8).

Participants reported smoking abstinence on an average of 28 (SD 31.43; 45%) days. Biochemically verified 7-day point prevalence smoking abstinence rates at follow-up were 20.6% (n=7), 17.6% (n=6), 26.5% (n=9), and 11.8% (n=4) at 4, 8, 12, and 26 weeks, respectively. Smoking status follow-up rates were 82.4% (n=28), 76.5% (n=26), 73.5% (n=25), and 64.7% (n=22) at 4, 8, 12, and 26 weeks post quit, respectively. Those who were missing at follow-up were considered smoking.

### Smoking Cessation Outcomes

#### VAR+NRT Versus VAR

As shown in Table 3, at each of the study follow-ups, biochemically verified smoking abstinence rates for those assigned to VAR+NRT were higher than those assigned to VAR alone. Table 4 provides descriptive data on daily smartphone assessments and shows that participants assigned to VAR+NRT reported ≈3 more days of abstinence than those assigned to VAR over the first 12 weeks post quit.

**Table 3:** Rates of biochemically verified 7-day point prevalence smoking abstinence at follow-up and overall MAQ^a^ medication adherence by treatment group.

	Reminder group			Medication group	
	REM^b^ (n=11), n (%)	NREM^c^ (n=23), n (%)	VAR^d^ (n=14), n (%)		VAR + NRT^e^ (n=20), n (%)
Week 4	3 (27)	4 (17)	2 (14)		5 (25)
Week 8	3 (27)	3 (13)	2 (14)		4 (20)
Week 12	6 (54)	3 (13)	3 (21)		6 (30)
Week 26	2 (18)	2 (9)	0 (0)		4 (20)
% High adherence (MAQ^f^) to varenicline during first 12 weeks postquit date	7 (64)	10 (44)	8 (57)		9 (45)

^a^MAQ: Medication Adherence Questionnaire.

^b^REM: reminder.

^c^NREM: no reminder.

^d^VAR: varenicline.

^e^NRT: nicotine replacement therapy.

^f^MAQ was considered high if score was median ≥3.60 (IQR 3.02-3.90).

**Table 4:** Smartphone-based measures of smoking cessation and medication adherence by treatment factor.

	Reminder group			Medication group	
	REM^a^	NREM^b^	VAR^c^		VAR + NRT^d^
NRT, pieces used per day, mean (SD)^e^	4.33 (2.50)	3.78 (6.80)	N/A^f^		4.10 (4.98)
NRT, days ≥5 pieces used, mean (SD)^e^	14.00 (15.57)	7.44 (9.80)	N/A		10.72 (13.06)
Varenicline, days adherent, mean (SD)^g^	58.55 (30.14)	40.52 (32.27)	50.71 (30.89)		43.30 (33.68)
Varenicline, proportion of days of adherent, n (%)^g^	0.20 (84)	0.30 (66)	0.23 (79)		0.31 (66)
Abstinent (days), mean (SD)^e^	40.45 (29.77)	21.77 (31.00)	26.29 (30.73)		29.26 (32.71)
Abstinent, proportion of days, n (%)^e^	0.33 (62)	0.42 (37)	0.41 (41)		0.42 (48)

^a^REM: reminder.

^b^NREM: no reminder.

^c^VAR: varenicline.

^d^NRT: nicotine replacement therapy.

^e^Assessed during the first 12 weeks after the scheduled quit date (84 days total).

^f^N/A: not applicable.

^g^Assessed from 1-week prequit (titration week) through the first 12 weeks after the scheduled quit date (91 days total).

#### REM Versus NREM

At study follow-ups, biochemically verified smoking abstinence rates for those assigned to REM were higher across all follow-ups than those assigned to the NREM group (Table 3). Those assigned to REM reported ≈19 more days of abstinence than those assigned to NREM over the first 12 weeks post quit.

### Medication Adherence

#### VAR+NRT Versus VAR

Participants assigned to VAR had higher average MAQ scores than those assigned to VAR+NRT (mean 3.52, SD 0.60 vs mean 2.97, SD 1.24). A higher proportion of those assigned to VAR reported high adherence on the MAQ compared with those assigned to VAR+NRT (Table 3). Smartphone-based assessments indicated the same pattern, with those assigned to VAR reporting ≈7 more days of varenicline adherence than those assigned to VAR+NRT (Table 4).

#### REM Versus NREM

Participants assigned to REM had higher average MAQ scores than those assigned to NREM (mean 3.41, SD 0.83 vs mean 3.10, SD 1.14). A higher proportion of participants assigned to REM reported high adherence on the MAQ compared to those who did not receive reminders (Table 3). Smartphone-based daily diaries indicated that participants assigned to REM reported ≈18 more days of varenicline adherence than NREM (Table 4). In the subsample of participants assigned to VAR+NRT, those assigned to REM group reported using a slightly higher average number of pieces of NRT per day compared to those in the NREM group. Further, those assigned to REM reported more days where they used ≥5 pieces of NRT per day than those assigned to NREM (Table 4).

#### Reasons for Nonadherence

Of the specified reasons for missing varenicline doses (n=276 instances throughout the entire study period), the most frequently reported reasons were side effects (70/258, 27.1%) and forgetting (36/258, 13.9%). Of the participants who reported varenicline side effects, 75.2% (328/436) reported that side effects were mild or very mild, with participants most commonly reporting sleep problems (307/574, 53.5%) or nausea (172/574, 30%). Likewise, of the participants who indicated that they did not use any NRT on a given day (702 instances across all participants throughout the study period), the most common reasons were “I did not feel like I needed it” (206/668, 30.8%) and “I did not have any with me” (95/668, 14.2%).

## Discussion

### Principal Findings

The purpose of this study was to evaluate the feasibility and potential efficacy of combined varenicline and oral NRT for smoking cessation and investigate the use of smartphone-based medication reminders to promote medication adherence and smoking cessation. Descriptive findings indicated that across all follow-ups, a greater percentage of participants assigned to receive combination varenicline and oral NRT were abstinent than those assigned to receive varenicline alone, although they showed poorer medication adherence. Participants who were assigned to the group that received medication reminders had higher smoking cessation rates across follow-ups, and they reported more days of abstinence, better medication adherence overall, more days of varenicline adherence, and more days where at least 5 pieces of NRT were used. Overall, preliminary findings support further investigation of the combination of varenicline with oral NRT for smoking cessation and smartphone-based medication reminders to promote medication adherence and smoking cessation.

Participants who were assigned to combined varenicline and oral NRT had higher rates of abstinence, which is supported by findings from meta-analyses indicating that varenicline combined with the nicotine patch was associated with higher rates of abstinence compared to varenicline alone [15,16]. Oral NRT offers users the ability to immediately address breakthrough cravings in comparison with the passive delivery of nicotine with the patch. Prior to this study, no studies had examined the combination of varenicline and oral NRT for smoking cessation in a randomized trial. However, multiple studies that have explored the efficacy of different combinations and doses of nicotine patches, other forms of NRT (gum and lozenges), and other smoking cessation medications (eg, bupropion and varenicline) have shown that combinations of pharmacotherapies often increase the likelihood of successful cessation [44-47]. Findings are also consistent with research demonstrating the feasibility and effectiveness of using medication reminders to promote smoking cessation medication adherence [21,22]. In a systematic review, Nieuwlaat et al [48] reported that medication adherence interventions using mobile devices were effective in improving medication adherence across a variety of conditions.

Previous research has shown that NRT adherence is strongly related to smoking cessation and may increase the likelihood of cessation by more than double [49]. For example, Ma et al [26] reported that wearing a nicotine patch for more hours per day and for more days in the first week following a quit attempt was associated with a greater likelihood of achieving smoking abstinence at follow-up among socioeconomically disadvantaged adults. Likewise, Hébert et al [25] reported that the odds of smoking on a given day were reduced with each additional piece of nicotine gum used [25]. Thus, practical and effective strategies are needed to improve medication adherence and cessation rates.

The most frequently reported causes of missed varenicline doses in this study were minor adverse reactions (sleep issues and nausea) and forgetfulness. The most frequent responses from people who did not take NRT on a particular day were “I did not feel like I needed it” and “I did not have any with me.” Other recent studies have indicated that NRT side effects and not believing NRT was effective were common reasons for nonadherence [25,49]. Thus, education regarding the benefits of smoking cessation pharmacotherapy (eg, medication increases the likelihood of successfully quitting and side effects may be time-limited), reminder messages, and minor adjustments to medication regimens or schedules (eg, medication timing and dose) may increase the likelihood of continued adherence.

### Limitations

A key limitation of this study was the small sample size. Due to the recall of varenicline in 2021, the study was discontinued before reaching the planned enrollment of 100 participants. Study analyses were not sufficiently powered to evaluate statistically significant differences between groups or support multilevel analyses. Nevertheless, descriptive analyses and initial study findings provide a starting point for future investigation.

### Future Directions

A full-scale randomized trial will be required before conclusions can be drawn about the efficacy of a combination of varenicline and oral NRT for smoking cessation. If combined varenicline and oral NRT are found to improve cessation rates over varenicline alone, adults who smoke will have a new evidence-based treatment option available to them as they undertake the difficult process of quitting smoking. However, medication regimens that include multiple medications and dosing multiple times per day may pose challenges to adherence [28,50]. Smartphone-based medication adherence interventions may be expanded to include multiple components in addition to reminders, such as education, dosing instructions, and novel smartphone-based medication refill requests [51]. Scheduled smartphone-based medication reminders have the potential to improve both adherence and smoking cessation outcomes. Smartphone interventions offer a flexible and scalable approach that can be adapted for other health conditions and medications.

## Appendix

Multimedia Appendix 1 CONSORT-eHEALTH checklist (V 1.6.1).

## Abbreviations

CO: carbon monoxide
MAQ: Medication Adherence Questionnaire
NREM: no reminder message
NRT: nicotine replacement therapy
REM: reminder message
REDCap: Research Electric Data Capture
TTRP: Tobacco Treatment Research Program
TTS: Tobacco Treatment Specialist
VAR: varenicline

## Data Availability

Data are available from the principal investigator (DEK) upon reasonable request (Darla-Kendzor@ouhsc.edu).
