# Supplementary material for: Varenicline Combined With Oral Nicotine Replacement Therapy and Smartphone-Based Medication Reminders for Smoking Cessation: Feasibility Randomized Controlled Trial
Source: JMIR Form Res. 2023 Oct 27;7:e48857. doi: 10.2196/48857 (PMC10638635; doi:10.2196/48857)
Supplement: Multimedia Appendix 1 [file formative_v7i1e48857_app1.pdf]

CONSORT-EHEALTH (V 1.6.1) - Submission/Publication Form

The CONSORT-EHEALTH checklist is intended for authors of randomized trials evaluating web-based and Internet-based applications/interventions, including mobile interventions, electronic games (incl multiplayer games), social media, certain telehealth applications, and other interactive and/or networked electronic applications. Some of the items (e.g. all subitems under item 5 - description of the intervention) may also be applicable for other study designs.

The goal of the CONSORT EHEALTH checklist and guideline is to be
a) a guide for reporting for authors of RCTs,
b) to form a basis for appraisal of an ehealth trial (in terms of validity)

CONSORT-EHEALTH items/subitems are MANDATORY reporting items for studies published in the Journal of Medical Internet Research and other journals / scientific societies endorsing the checklist.

Items numbered 1., 2., 3., 4a., 4b etc are original CONSORT or CONSORT-NPT (non-pharmacologic treatment) items.
Items with Roman numerals (i., ii, iii, iv etc.) are CONSORT-EHEALTH extensions/clarifications.

As the CONSORT-EHEALTH checklist is still considered in a formative stage, we would ask that you also RATE ON A SCALE OF 1-5 how important/useful you feel each item is FOR THE PURPOSE OF THE CHECKLIST and reporting guideline (optional).

Mandatory reporting items are marked with a red *.
In the textboxes, either copy & paste the relevant sections from your manuscript into this form - please include any quotes from your manuscript in QUOTATION MARKS,
or answer directly by providing additional information not in the manuscript, or elaborating on why the item was not relevant for this study.

YOUR ANSWERS WILL BE PUBLISHED AS A SUPPLEMENTARY FILE TO YOUR PUBLICATION IN JMIR AND ARE CONSIDERED PART OF YOUR PUBLICATION (IF ACCEPTED).
Please fill in these questions diligently. Information will not be copyedited, so please use proper spelling and grammar, use correct capitalization, and avoid abbreviations.

DO NOT FORGET TO SAVE AS PDF _AND_ CLICK THE SUBMIT BUTTON SO YOUR ANSWERS ARE IN OUR DATABASE !!!

Your response is too large. Try shortening some answers.

Eysenbach G, CONSORT-EHEALTH Group

CONSORT-EHEALTH: Improving and Standardizing Evaluation Reports of Web-based and Mobile Health Interventions
J Med Internet Res 2011;13(4):e126
URL: http://www.jmir.org/2011/4/e126/
doi: 10.2196/jmir.1923
PMID: 22209829

munjireen.sifat@gmail.com Switch account

Not shared

* Indicates required question

Your name *

First Last

Munjireen Sifat

Primary Affiliation (short), City, Country *

University of Toronto, Toronto, Canada

University of Oklahoma, Oklahoma City, USA

Your e-mail address *

abc@gmail.com

mys519@jefferson.edu

Your response is too large. Try shortening some answers.

Title of your manuscript *

Provide the (draft) title of your manuscript.

Varenicline Combined With Oral Nicotine Replacement Therapy and Smartphone-Based Medication Reminders for Smoking Cessation: Feasibility Randomized Controlled Trial

Name of your App/Software/Intervention *

If there is a short and a long/alternate name, write the short name first and add the long name in brackets.

INSIGHT

Evaluated Version (if any)

e.g. "V1", "Release 2017-03-01", "Version 2.0.27913"

Your answer

Language(s) *

What language is the intervention/app in? If multiple languages are available, separate by comma (e.g. "English, French")

English, Lao

URL of your Intervention Website or App

e.g. a direct link to the mobile app on app in appstore (itunes, Google Play), or URL of the website. If the intervention is a DVD or hardware, you can also link to an Amazon page.

https://healthpromotionresearch.org/Mobile-Health-Technology

Your response is too large. Try shortening some answers.

URL of an image/screenshot (optional)

Your answer

Accessibility *

Can an enduser access the intervention presently?

- ( ) access is free and open
- ( ) access only for special usergroups, not open
- ( ) access is open to everyone, but requires payment/subscription/in-app purchases
- (x) app/intervention no longer accessible
- ( ) Other:

Primary Medical Indication/Disease/Condition *

e.g. "Stress", "Diabetes", or define the target group in brackets after the condition, e.g. "Autism (Parents of children with)", "Alzheimers (Informal Caregivers of)"

smoking cessation

Primary Outcomes measured in trial *

comma-separated list of primary outcomes reported in the trial

smoking cessation, medication adherence

Your response is too large. Try shortening some answers.

Secondary/other outcomes

Are there any other outcomes the intervention is expected to affect?

Your answer

Recommended "Dose" *

What do the instructions for users say on how often the app should be used?

- (x) Approximately Daily
- ( ) Approximately Weekly
- ( ) Approximately Monthly
- ( ) Approximately Yearly
- ( ) "as needed"
- ( ) Other:

Your response is too large. Try shortening some answers.

Approx. Percentage of Users (starters) still using the app as recommended after 3 months *

- ○ unknown / not evaluated
- ○ 0-10%
- ○ 11-20%
- ○ 21-30%
- ○ 31-40%
- ○ 41-50%
- ○ 51-60%
- ○ 61-70%
- ○ 71%-80%
- ○ 81-90%
- ○ 91-100%
- ◉ Other: Intervention was meant to be only 12 weeks

Overall, was the app/intervention effective? *

- ○ yes: all primary outcomes were significantly better in intervention group vs control
- ○ partly: SOME primary outcomes were significantly better in intervention group vs control
- ○ no statistically significant difference between control and intervention
- ○ potentially harmful: control was significantly better than intervention in one or more outcomes
- ○ inconclusive: more research is needed

Your response is too large. Try shortening some answers.

Article Preparation Status/Stage *

At which stage in your article preparation are you currently (at the time you fill in this form)

- ○ not submitted yet - in early draft status
- ○ not submitted yet - in late draft status, just before submission
- ○ submitted to a journal but not reviewed yet
- ○ submitted to a journal and after receiving initial reviewer comments
- ● submitted to a journal and accepted, but not published yet
- ○ published
- ○ Other:

Journal *

If you already know where you will submit this paper (or if it is already submitted), please provide the journal name (if it is not JMIR, provide the journal name under "other")

- ○ not submitted yet / unclear where I will submit this
- ○ Journal of Medical Internet Research (JMIR)
- ○ JMIR mHealth and UHealth
- ○ JMIR Serious Games
- ○ JMIR Mental Health
- ○ JMIR Public Health
- ● JMIR Formative Research
- ○ Other JMIR sister journal
- ○ Other:

Your response is too large. Try shortening some answers.

Is this a full powered effectiveness trial or a pilot/feasibility trial? *

- (selected) Pilot/feasibility
- Fully powered

Manuscript tracking number *

If this is a JMIR submission, please provide the manuscript tracking number under "other" (The ms tracking number can be found in the submission acknowledgement email, or when you login as author in JMIR. If the paper is already published in JMIR, then the ms tracking number is the four-digit number at the end of the DOI, to be found at the bottom of each published article in JMIR)

- no ms number (yet) / not (yet) submitted to / published in JMIR
- (selected) Other: #48857

TITLE AND ABSTRACT

1a) TITLE: Identification as a randomized trial in the title

1a) Does your paper address CONSORT item 1a? *

I.e does the title contain the phrase "Randomized Controlled Trial"? (if not, explain the reason under "other")

- (selected) yes
- Other:

Your response is too large. Try shortening some answers.

1a-i) Identify the mode of delivery in the title

Identify the mode of delivery. Preferably use “web-based” and/or “mobile” and/or “electronic game” in the title. Avoid ambiguous terms like “online”, “virtual”, “interactive”. Use “Internet-based” only if Intervention includes non-web-based Internet components (e.g. email), use “computer-based” or “electronic” only if offline products are used. Use “virtual” only in the context of “virtual reality” (3-D worlds). Use “online” only in the context of “online support groups”. Complement or substitute product names with broader terms for the class of products (such as “mobile” or “smart phone” instead of “iphone”), especially if the application runs on different platforms.

subitem not at all important

- 1 ○
- 2 ○
- 3 ◉
- 4 ○
- 5 ○

essential

Clear selection

Does your paper address subitem 1a-i? *

Copy and paste relevant sections from manuscript title (include quotes in quotation marks "like this" to indicate direct quotes from your manuscript), or elaborate on this item by providing additional information not in the ms, or briefly explain why the item is not applicable/relevant for your study

Varenicline Combined With Oral Nicotine Replacement Therapy and Smartphone-Based Medication Reminders for Smoking Cessation: Feasibility Randomized Controlled Trial

Your response is too large. Try shortening some answers.

1a-ii) Non-web-based components or important co-interventions in title

Mention non-web-based components or important co-interventions in title, if any (e.g., “with telephone support”).

subitem not at all important

- 1 ○
- 2 ○
- 3 ○
- 4 ○
- 5 ◉

essential

Clear selection

Does your paper address subitem 1a-ii?

Copy and paste relevant sections from manuscript title (include quotes in quotation marks "like this" to indicate direct quotes from your manuscript), or elaborate on this item by providing additional information not in the ms, or briefly explain why the item is not applicable/relevant for your study

"Varenicline Combined With Oral Nicotine Replacement Therapy" and Smartphone-Based Medication Reminders for Smoking Cessation: Feasibility Randomized Controlled Trial

Your response is too large. Try shortening some answers.

1a-iii) Primary condition or target group in the title

Mention primary condition or target group in the title, if any (e.g., “for children with Type I Diabetes”) Example: A Web-based and Mobile Intervention with Telephone Support for Children with Type I Diabetes: Randomized Controlled Trial

subitem not at all important

1 ○

2 ○

3 ○

4 ○

5 ◉

essential

Clear selection

Does your paper address subitem 1a-iii? *

Copy and paste relevant sections from manuscript title (include quotes in quotation marks "like this" to indicate direct quotes from your manuscript), or elaborate on this item by providing additional information not in the ms, or briefly explain why the item is not applicable/relevant for your study

Varenicline Combined With Oral Nicotine Replacement Therapy and Smartphone-Based Medication Reminders "for Smoking Cessation": Feasibility Randomized Controlled Trial

1b) ABSTRACT: Structured summary of trial design, methods, results, and conclusions

NPT extension: Description of experimental treatment, comparator, care providers, centers, and blinding status.

Your response is too large. Try shortening some answers.

1b-i) Key features/functionalities/components of the intervention and comparator in the METHODS section of the ABSTRACT

Mention key features/functionalities/components of the intervention and comparator in the abstract. If possible, also mention theories and principles used for designing the site. Keep in mind the needs of systematic reviewers and indexers by including important synonyms. (Note: Only report in the abstract what the main paper is reporting. If this information is missing from the main body of text, consider adding it)

subitem not at all important

1 ○

2 ○

3 ○

4 ○

5 ◉

essential

Clear selection

Does your paper address subitem 1b-i? *

Copy and paste relevant sections from the manuscript abstract (include quotes in quotation marks "like this" to indicate direct quotes from your manuscript), or elaborate on this item by providing additional information not in the ms, or briefly explain why the item is not applicable/relevant for your study

A 2×2 factorial design was used. Participants (N=34) were randomized to (1) varenicline + oral NRT (VAR+NRT) or varenicline alone (VAR) and (2) smartphone medication reminder messages (REM) or no reminder messages (NREM) over 13 weeks. Participants assigned to VAR+REM received varenicline reminder prompts, and those assigned to VAR+NRT+REM also received reminders to use oral NRT. The other 2 groups (VAR+NREM and VAR+NRT+NREM) did not receive medication reminders. All participants received tobacco cessation counseling.

Your response is too large. Try shortening some answers.

1b-ii) Level of human involvement in the METHODS section of the ABSTRACT

Clarify the level of human involvement in the abstract, e.g., use phrases like “fully automated” vs. “therapist/nurse/care provider/physician-assisted” (mention number and expertise of providers involved, if any). (Note: Only report in the abstract what the main paper is reporting. If this information is missing from the main body of text, consider adding it)

subitem not at all important

1 ○

2 ○

3 ○

4 ◉

5 ○

essential

Clear selection

Does your paper address subitem 1b-ii?

Copy and paste relevant sections from the manuscript abstract (include quotes in quotation marks "like this" to indicate direct quotes from your manuscript), or elaborate on this item by providing additional information not in the ms, or briefly explain why the item is not applicable/relevant for your study

All participants received tobacco cessation counseling.

Your response is too large. Try shortening some answers.

1b-iii) Open vs. closed, web-based (self-assessment) vs. face-to-face assessments in the METHODS section of the ABSTRACT

Mention how participants were recruited (online vs. offline), e.g., from an open access website or from a clinic or a closed online user group (closed usergroup trial), and clarify if this was a purely web-based trial, or there were face-to-face components (as part of the intervention or for assessment). Clearly say if outcomes were self-assessed through questionnaires (as common in web-based trials). Note: In traditional offline trials, an open trial (open-label trial) is a type of clinical trial in which both the researchers and participants know which treatment is being administered. To avoid confusion, use "blinded" or "unblinded" to indicated the level of blinding instead of "open", as "open" in web-based trials usually refers to "open access" (i.e. participants can self-enrol). (Note: Only report in the abstract what the main paper is reporting. If this information is missing from the main body of text, consider adding it)

subitem not at all important

1 ○

2 ○

3 ◉

4 ○

5 ○

essential

Clear selection

Does your paper address subitem 1b-iii?

Copy and paste relevant sections from the manuscript abstract (include quotes in quotation marks "like this" to indicate direct quotes from your manuscript), or elaborate on this item by providing additional information not in the ms, or briefly explain why the item is not applicable/relevant for your study

"Participants were not blinded to intervention groups. "

Your response is too large. Try shortening some answers.

1b-iv) RESULTS section in abstract must contain use data

Report number of participants enrolled/assessed in each group, the use/uptake of the intervention (e.g., attrition/adherence metrics, use over time, number of logins etc.), in addition to primary/secondary outcomes. (Note: Only report in the abstract what the main paper is reporting. If this information is missing from the main body of text, consider adding it)

subitem not at all important

1 ○
2 ○
3 ◉
4 ○
5 ○

essential

Clear selection

Does your paper address subitem 1b-iv?

Copy and paste relevant sections from the manuscript abstract (include quotes in quotation marks "like this" to indicate direct quotes from your manuscript), or elaborate on this item by providing additional information not in the ms, or briefly explain why the item is not applicable/relevant for your study

No - given this was a pilot study with a small sample size the focus was on the main outcomes.

Your response is too large. Try shortening some answers.

1b-v) CONCLUSIONS/DISCUSSION in abstract for negative trials

Conclusions/Discussions in abstract for negative trials: Discuss the primary outcome - if the trial is negative (primary outcome not changed), and the intervention was not used, discuss whether negative results are attributable to lack of uptake and discuss reasons. (Note: Only report in the abstract what the main paper is reporting. If this information is missing from the main body of text, consider adding it)

subitem not at all important

1 ○

2 ○

3 ◉

4 ○

5 ○

essential

Clear selection

Does your paper address subitem 1b-v?

Copy and paste relevant sections from the manuscript abstract (include quotes in quotation marks "like this" to indicate direct quotes from your manuscript), or elaborate on this item by providing additional information not in the ms, or briefly explain why the item is not applicable/relevant for your study

N/A this is not a negative trial

INTRODUCTION

2a) In INTRODUCTION: Scientific background and explanation of rationale

Your response is too large. Try shortening some answers.

2a-i) Problem and the type of system/solution

Describe the problem and the type of system/solution that is object of the study: intended as stand-alone intervention vs. incorporated in broader health care program? Intended for a particular patient population? Goals of the intervention, e.g., being more cost-effective to other interventions, replace or complement other solutions? (Note: Details about the intervention are provided in "Methods" under 5)

subitem not at all important

1 ○

2 ○

3 ◉

4 ○

5 ○

essential

Clear selection

Does your paper address subitem 2a-i? *

Copy and paste relevant sections from the manuscript (include quotes in quotation marks "like this" to indicate direct quotes from your manuscript), or elaborate on this item by providing additional information not in the ms, or briefly explain why the item is not applicable/relevant for your study

Smoking is the leading modifiable cause of cancer and cancer mortality in the United States [1]. Although the prevalence of smoking has declined over the past 5 decades, approximately 12.5% of adults continue to smoke in the United States [2]. Smoking is known to cause nearly 20% of all cancers, 30% of cancer deaths [1], and approximately 80% of lung cancers [3]. Smoking cessation increases life expectancy, and quitting at an earlier age is associated with more years of life gained than quitting later in life [4]. Effective pharmacotherapies to aid cessation are available, and the combination of behavioral support with pharmacotherapy is associated with optimal cessation outcomes [5]. More recently, research has focused on evaluating combination pharmacotherapies in an effort to refine current evidence-based treatment approaches.

Your response is too large. Try shortening some answers.

2a-ii) Scientific background, rationale: What is known about the (type of) system

Scientific background, rationale: What is known about the (type of) system that is the object of the study (be sure to discuss the use of similar systems for other conditions/diagnoses, if appropiate), motivation for the study, i.e. what are the reasons for and what is the context for this specific study, from which stakeholder viewpoint is the study performed, potential impact of findings [2]. Briefly justify the choice of the comparator.

subitem not at all important

1 ○

2 ○

3 ◉

4 ○

5 ○

essential

Clear selection

Your response is too large. Try shortening some answers.

Does your paper address subitem 2a-ii? *

Copy and paste relevant sections from the manuscript (include quotes in quotation marks "like this" to indicate direct quotes from your manuscript), or elaborate on this item by providing additional information not in the ms, or briefly explain why the item is not applicable/relevant for your study

Varenicline is considered a first-line tobacco cessation treatment [6]. Varenicline and nicotine replacement therapy (NRT) each promote smoking cessation via potentially complementary pathways. Varenicline is a partial nicotine receptor agonist and also has nicotine receptor antagonist properties. The agonist effect of varenicline is about half the effect of nicotine at the receptor, thereby reducing craving and withdrawal during periods of smoking abstinence while also competing with nicotine for binding at the receptor site, thereby making smoking less reinforcing [7,8]. The nicotine in lozenges is a full nicotine receptor agonist, thus, reducing craving and withdrawal similar to nicotine in cigarettes [9]. However, the slower absorption of nicotine in lozenges through the buccal mucosa (vs the lungs with cigarette smoking) along with the absence of other additive and addictive compounds (eg, acetaldehyde and menthol) reduces the addiction potential of oral NRT [9]. Since varenicline is a partial agonist and may not fully saturate nicotine receptors, NRT can supplement the effects of varenicline both preemptively and during acute cravings [10].

2b) In INTRODUCTION: Specific objectives or hypotheses

Does your paper address CONSORT subitem 2b? *

Copy and paste relevant sections from the manuscript (include quotes in quotation marks "like this" to indicate direct quotes from your manuscript), or elaborate on this item by providing additional information not in the ms, or briefly explain why the item is not applicable/relevant for your study

The purpose of this pilot study was to characterize the feasibility and preliminary efficacy of a combination of varenicline and oral NRT among adults initiating smoking cessation treatment. In addition, this study described the potential impact of smartphone-based medication reminder prompts on medication adherence and smoking cessation. Findings will provide initial information regarding the possible benefits of a combination of varenicline and oral NRT and smartphone medication reminders and inform decisions about whether further investigation is warranted.

Your response is too large. Try shortening some answers.

METHODS

3a) Description of trial design (such as parallel, factorial) including allocation ratio

Does your paper address CONSORT subitem 3a? *

Copy and paste relevant sections from the manuscript (include quotes in quotation marks "like this" to indicate direct quotes from your manuscript), or elaborate on this item by providing additional information not in the ms, or briefly explain why the item is not applicable/relevant for your study

This study used a 2×2 factorial design (Table 1). Participants (N=34) were randomized to (1) varenicline + nicotine gum or lozenges (VAR+NRT; n=20) or varenicline alone (VAR; n=14) and (2) smartphone medication reminder messages (REM; n=11) or no reminder messages (NREM; n=23). Adaptive randomization [37,38] was used to assign participants to groups based on race, sex, cigarettes smoked per day, and education. Participants assigned to VAR+REM (n=2) received varenicline reminder prompts, and those assigned to VAR+NRT+REM (n=9) also received reminders to use gum or lozenges with decreasing frequency over time. Participants assigned to VAR+NREM (n=12) and VAR+NRT+NREM (n=11) did not receive medication reminders. Participants were followed for 27 weeks, beginning 1 week before their scheduled quit date through 26 weeks after their quit date, with key follow-up assessments of smoking status at 4, 8, 12, and 26 weeks postquit. Participants also completed daily smartphone assessments from 1 week before their quit date through 12 weeks after their quit date. All participants were enrolled between January 2020 and July 2021, and final 26-week follow-up visits were completed by February 2022.

3b) Important changes to methods after trial commencement (such as eligibility criteria), with reasons

Your response is too large. Try shortening some answers.

Does your paper address CONSORT subitem 3b? *

Copy and paste relevant sections from the manuscript (include quotes in quotation marks "like this" to indicate direct quotes from your manuscript), or elaborate on this item by providing additional information not in the ms, or briefly explain why the item is not applicable/relevant for your study

The study was discontinued early due to the Pfizer recall of varenicline (Chantix) following findings that nitrosamine levels were above the U.S. Food and Drug Administration [39] acceptable intake limit. However, note that generic varenicline has become available since the conclusion of the study and can be accessed in future research

3b-i) Bug fixes, Downtimes, Content Changes

Bug fixes, Downtimes, Content Changes: ehealth systems are often dynamic systems. A description of changes to methods therefore also includes important changes made on the intervention or comparator during the trial (e.g., major bug fixes or changes in the functionality or content) (5-iii) and other “unexpected events” that may have influenced study design such as staff changes, system failures/downtimes, etc. [2].

subitem not at all important

- 1 ○
- 2 ○
- 3 ◉
- 4 ○
- 5 ○

essential

Clear selection

Your response is too large. Try shortening some answers.

Does your paper address subitem 3b-i?

Copy and paste relevant sections from the manuscript (include quotes in quotation marks "like this" to indicate direct quotes from your manuscript), or elaborate on this item by providing additional information not in the ms, or briefly explain why the item is not applicable/relevant for your study

N/A we did not encounter these issues

4a) Eligibility criteria for participants

Does your paper address CONSORT subitem 4a? *

Copy and paste relevant sections from the manuscript (include quotes in quotation marks "like this" to indicate direct quotes from your manuscript), or elaborate on this item by providing additional information not in the ms, or briefly explain why the item is not applicable/relevant for your study

Individuals were eligible for the study if they (1) were ≥18 years of age, (2) demonstrated higher than sixth-grade English literacy level (assessed via the Short-Form Rapid Estimate of Adult Literacy in Medicine [34]), (3) agreed to install the study smartphone app onto their personal phone or were willing to carry a study-provided phone with the app, (4) had an expired carbon monoxide (CO) level >6 ppm suggestive of current smoking [35], (5) reported smoking ≥5 cigarettes per day at the time of enrollment, (6) were willing to initiate a quit attempt ≈7 days after enrollment, (7) were willing to use nicotine gum or lozenges, and (8) were eligible to use varenicline after consultation with the study physician. Individuals were excluded from the study if they (1) had a history of seizures, (2) were allergic to varenicline, (3) were considered to be at high risk for suicide (score ≥10 on the Mini-International Neuropsychiatric Interview Suicidal Scale [36]), or (4) were pregnant, planning to become pregnant, or currently breastfeeding at the time of enrollment.

Your response is too large. Try shortening some answers.

4a-i) Computer / Internet literacy

Computer / Internet literacy is often an implicit “de facto” eligibility criterion - this should be explicitly clarified.

subitem not at all important

1 ○

2 ○

3 ◉

4 ○

5 ○

essential

Clear selection

Does your paper address subitem 4a-i?

Copy and paste relevant sections from the manuscript (include quotes in quotation marks "like this" to indicate direct quotes from your manuscript), or elaborate on this item by providing additional information not in the ms, or briefly explain why the item is not applicable/relevant for your study

We did not assess digital literacy, but instead assessed (2) demonstrated higher than sixth-grade English literacy level (assessed via the Short-Form Rapid Estimate of Adult Literacy in Medicine [34]). Participants were able to call members of the research team for digital help.

Your response is too large. Try shortening some answers.

4a-ii) Open vs. closed, web-based vs. face-to-face assessments:

Open vs. closed, web-based vs. face-to-face assessments: Mention how participants were recruited (online vs. offline), e.g., from an open access website or from a clinic, and clarify if this was a purely web-based trial, or there were face-to-face components (as part of the intervention or for assessment), i.e., to what degree got the study team to know the participant. In online-only trials, clarify if participants were quasi-anonymous and whether having multiple identities was possible or whether technical or logistical measures (e.g., cookies, email confirmation, phone calls) were used to detect/prevent these.

subitem not at all important

1 ○

2 ○

3 ◉

4 ○

5 ○

essential

Clear selection

Your response is too large. Try shortening some answers.

Does your paper address subitem 4a-ii? *

Copy and paste relevant sections from the manuscript (include quotes in quotation marks "like this" to indicate direct quotes from your manuscript), or elaborate on this item by providing additional information not in the ms, or briefly explain why the item is not applicable/relevant for your study

Participants were asked to complete web-based assessments via the Research Electric Data Capture program (REDCap; Vanderbilt University [40,41]) at baseline (1 week prequit) and then weekly through 4 weeks postquit with additional follow-up assessments at 8, 12, and 26 weeks postquit. Participants were compensated US $20 for each completed assessment (up to US $120).
Participants were prompted via the Insight smartphone app [42] to complete daily diary assessments 30 minutes after their self-reported usual wake time. Daily smoking, medication adherence, and other relevant variables were assessed from 1 week prior to the scheduled quit date through 12 weeks postquit. Participants also received medication reminder prompts (described in the Medication REM section). Those who responded that they did not take their medication as prescribed were asked about their reasons for nonadherence. Participants were compensated up to US $10 per week for the completion of daily diary assessments (up to US $130 total). Specifically, participants earned US $10 for completing 6-7 daily diary assessments or US $5 for completing 4-5 assessments each week. Participants who completed ≤3 daily diary assessments did not receive compensation for that week. The primary study outcomes were CO-confirmed, self-reported 7-day point prevalence abstinence at 4, 8, 12, and 26 weeks postquit follow-up.
Participants were initially asked to complete study follow-up assessments in person at the TTRP, but the onset of the COVID-19 pandemic led the university to limit in-person interactions on campus. As a result, between March and May 2020, all assessments were completed remotely via web-based assessments, daily smartphone assessments, and smartphone-based breath sample submissions. After the university reopened, participants were encouraged to attend study visits in person, but remote assessments were accommodated.

Your response is too large. Try shortening some answers.

4a-iii) Information giving during recruitment

Information given during recruitment. Specify how participants were briefed for recruitment and in the informed consent procedures (e.g., publish the informed consent documentation as appendix, see also item X26), as this information may have an effect on user self-selection, user expectation and may also bias results.

subitem not at all important

1 ○

2 ○

3 ◉

4 ○

5 ○

essential

Clear selection

Does your paper address subitem 4a-iii?

Copy and paste relevant sections from the manuscript (include quotes in quotation marks "like this" to indicate direct quotes from your manuscript), or elaborate on this item by providing additional information not in the ms, or briefly explain why the item is not applicable/relevant for your study

Counseling
Approximately 1-week prior to the scheduled quit date, a Tobacco Treatment Specialist (TTS) provided an overview of the tobacco cessation program and assisted participants with developing a quit plan. Participants were instructed to quit smoking at bedtime or 10 PM (whichever occurred first) on the evening before their next weekly counseling session (≈1 week after enrollment). All participants were offered up to 5 additional weekly counseling sessions delivered in-person or by telephone with a TTS. Topics of discussion during the counseling sessions included (1) the impact of tobacco on the health benefits of quitting, (2) stress management strategies, (3) making positive lifestyle changes, (4) developing coping skills, and (5) relapse prevention. The TTS checked in with participants each week about the difficulties and successes they experienced and planned for anticipated future challenges.

Your response is too large. Try shortening some answers.

4b) Settings and locations where the data were collected

Does your paper address CONSORT subitem 4b? *

Copy and paste relevant sections from the manuscript (include quotes in quotation marks "like this" to indicate direct quotes from your manuscript), or elaborate on this item by providing additional information not in the ms, or briefly explain why the item is not applicable/relevant for your study

Data was primarily self-report at the participants location of convenience.

4b-i) Report if outcomes were (self-)assessed through online questionnaires

Clearly report if outcomes were (self-)assessed through online questionnaires (as common in web-based trials) or otherwise.

subitem not at all important

- 1 ○
- 2 ○
- 3 ◉
- 4 ○
- 5 ○

essential

Clear selection

Your response is too large. Try shortening some answers.

Does your paper address subitem 4b-i? *

Copy and paste relevant sections from the manuscript (include quotes in quotation marks "like this" to indicate direct quotes from your manuscript), or elaborate on this item by providing additional information not in the ms, or briefly explain why the item is not applicable/relevant for your study

Participants were asked to complete web-based assessments via the Research Electric Data Capture program (REDCap; Vanderbilt University [40,41]) at baseline (1 week prequit) and then weekly through 4 weeks postquit with additional follow-up assessments at 8, 12, and 26 weeks postquit. Participants were compensated US $20 for each completed assessment (up to US $120).
Participants were prompted via the Insight smartphone app [42] to complete daily diary assessments 30 minutes after their self-reported usual wake time. Daily smoking, medication adherence, and other relevant variables were assessed from 1 week prior to the scheduled quit date through 12 weeks postquit. Participants also received medication reminder prompts (described in the Medication REM section). Those who responded that they did not take their medication as prescribed were asked about their reasons for nonadherence. Participants were compensated up to US $10 per week for the completion of daily diary assessments (up to US $130 total). Specifically, participants earned US $10 for completing 6-7 daily diary assessments or US $5 for completing 4-5 assessments each week. Participants who completed ≤3 daily diary assessments did not receive compensation for that week. The primary study outcomes were CO-confirmed, self-reported 7-day point prevalence abstinence at 4, 8, 12, and 26 weeks postquit follow-up.
Participants were initially asked to complete study follow-up assessments in person at the TTRP, but the onset of the COVID-19 pandemic led the university to limit in-person interactions on campus. As a result, between March and May 2020, all assessments were completed remotely via web-based assessments, daily smartphone assessments, and smartphone-based breath sample submissions. After the university reopened, participants were encouraged to attend study visits in person, but remote assessments

Your response is too large. Try shortening some answers.

4b-ii) Report how institutional affiliations are displayed

Report how institutional affiliations are displayed to potential participants [on ehealth media], as affiliations with prestigious hospitals or universities may affect volunteer rates, use, and reactions with regards to an intervention.(Not a required item – describe only if this may bias results)

subitem not at all important

1 ○

2 ○

3 ◉

4 ○

5 ○

essential

Clear selection

Your response is too large. Try shortening some answers.

Does your paper address subitem 4b-ii?

Copy and paste relevant sections from the manuscript (include quotes in quotation marks "like this" to indicate direct quotes from your manuscript), or elaborate on this item by providing additional information not in the ms, or briefly explain why the item is not applicable/relevant for your study

1 TSET Health Promotion Research Center, Stephenson Cancer Center, University of Oklahoma Health Sciences Center, Oklahoma City, OK
2 The University of Texas Health Science Center, School of Public Health, Austin, TX
3 Department of Behavioral and Social Sciences, Brown University School of Public Health, Providence, RI
4 Department of Family and Preventive Medicine, University of Oklahoma Health Sciences Center, Oklahoma City, OK
5 Johns Hopkins Bloomberg School of Public Health, Baltimore, MD
6 Hudson College of Public Health, Department of Biostatistics and Epidemiology
University of Oklahoma Health Sciences Center, Oklahoma City, OK
7 Department of Pharmacy: Clinical and Administrative Sciences
University of Oklahoma Health Sciences Center, Oklahoma City, OK
8 Department of Psychiatry and Behavioral Sciences
University of Oklahoma Health Sciences Center, Oklahoma City, OK

5) The interventions for each group with sufficient details to allow replication, including how and when they were actually administered

Your response is too large. Try shortening some answers.

5-i) Mention names, credential, affiliations of the developers, sponsors, and owners

Mention names, credential, affiliations of the developers, sponsors, and owners [6] (if authors/evaluators are owners or developer of the software, this needs to be declared in a "Conflict of interest" section or mentioned elsewhere in the manuscript).

subitem not at all important

1 ○

2 ○

3 ◉

4 ○

5 ○

essential

Clear selection

Does your paper address subitem 5-i?

Copy and paste relevant sections from the manuscript (include quotes in quotation marks "like this" to indicate direct quotes from your manuscript), or elaborate on this item by providing additional information not in the ms, or briefly explain why the item is not applicable/relevant for your study

Conflicts of Interest
MSB and DEK are inventors of the Insight mHealth Platform, which was used to develop the study app. They receive royalties related to its use. However, they did not receive royalties in this case.

Your response is too large. Try shortening some answers.

5-ii) Describe the history/development process

Describe the history/development process of the application and previous formative evaluations (e.g., focus groups, usability testing), as these will have an impact on adoption/use rates and help with interpreting results.

subitem not at all important

1 ○

2 ○

3 ◉

4 ○

5 ○

essential

Clear selection

Does your paper address subitem 5-ii?

Copy and paste relevant sections from the manuscript (include quotes in quotation marks "like this" to indicate direct quotes from your manuscript), or elaborate on this item by providing additional information not in the ms, or briefly explain why the item is not applicable/relevant for your study

The app developer website is referenced, Participants were prompted via the Insight smartphone app [42] to complete daily diary assessments 30 minutes after their self-reported usual wake time.

Your response is too large. Try shortening some answers.

5-iii) Revisions and updating

Revisions and updating. Clearly mention the date and/or version number of the application/intervention (and comparator, if applicable) evaluated, or describe whether the intervention underwent major changes during the evaluation process, or whether the development and/or content was “frozen” during the trial. Describe dynamic components such as news feeds or changing content which may have an impact on the replicability of the intervention (for unexpected events see item 3b).

subitem not at all important

1 ○

2 ○

3 ◉

4 ○

5 ○

essential

Clear selection

Does your paper address subitem 5-iii?

Copy and paste relevant sections from the manuscript (include quotes in quotation marks "like this" to indicate direct quotes from your manuscript), or elaborate on this item by providing additional information not in the ms, or briefly explain why the item is not applicable/relevant for your study

Not included as the intervention is not yet available publicly and we plan to have a larger scale trial first.

Your response is too large. Try shortening some answers.

5-iv) Quality assurance methods

Provide information on quality assurance methods to ensure accuracy and quality of information provided [1], if applicable.

subitem not at all important

- 1 ○
- 2 ○
- 3 ◉
- 4 ○
- 5 ○

essential

Clear selection

Does your paper address subitem 5-iv?

Copy and paste relevant sections from the manuscript (include quotes in quotation marks "like this" to indicate direct quotes from your manuscript), or elaborate on this item by providing additional information not in the ms, or briefly explain why the item is not applicable/relevant for your study

Not included as the intervention is a feasibility trial and we plan to have a larger scale trial first.

Your response is too large. Try shortening some answers.

5-v) Ensure replicability by publishing the source code, and/or providing screenshots/screen-capture video, and/or providing flowcharts of the algorithms used

Ensure replicability by publishing the source code, and/or providing screenshots/screen-capture video, and/or providing flowcharts of the algorithms used. Replicability (i.e., other researchers should in principle be able to replicate the study) is a hallmark of scientific reporting.

subitem not at all important

- 1 ○
- 2 ○
- 3 ◉
- 4 ○
- 5 ○

essential

Clear selection

Does your paper address subitem 5-v?

Copy and paste relevant sections from the manuscript (include quotes in quotation marks "like this" to indicate direct quotes from your manuscript), or elaborate on this item by providing additional information not in the ms, or briefly explain why the item is not applicable/relevant for your study

Not included as the intervention is not yet available publicly and we plan to have a larger scale trial first.

Your response is too large. Try shortening some answers.

5-vi) Digital preservation

Digital preservation: Provide the URL of the application, but as the intervention is likely to change or disappear over the course of the years; also make sure the intervention is archived (Internet Archive, webcitation.org, and/or publishing the source code or screenshots/videos alongside the article). As pages behind login screens cannot be archived, consider creating demo pages which are accessible without login.

subitem not at all important

1 ○

2 ○

3 ◉

4 ○

5 ○

essential

Clear selection

Does your paper address subitem 5-vi?

Copy and paste relevant sections from the manuscript (include quotes in quotation marks "like this" to indicate direct quotes from your manuscript), or elaborate on this item by providing additional information not in the ms, or briefly explain why the item is not applicable/relevant for your study

Not included as the intervention is not yet available publicly and we plan to have a larger scale trial first. However, a reference to the website that details the App is provided.

Your response is too large. Try shortening some answers.

5-vii) Access

Access: Describe how participants accessed the application, in what setting/context, if they had to pay (or were paid) or not, whether they had to be a member of specific group. If known, describe how participants obtained “access to the platform and Internet” [1]. To ensure access for editors/reviewers/readers, consider to provide a “backdoor” login account or demo mode for reviewers/readers to explore the application (also important for archiving purposes, see vi).

subitem not at all important

1 ○

2 ○

3 ◉

4 ○

5 ○

essential

Clear selection

Does your paper address subitem 5-vii? *

Copy and paste relevant sections from the manuscript (include quotes in quotation marks "like this" to indicate direct quotes from your manuscript), or elaborate on this item by providing additional information not in the ms, or briefly explain why the item is not applicable/relevant for your study

Participants were prompted via the Insight smartphone app [42] to complete daily diary assessments 30 minutes after their self-reported usual wake time. Daily smoking, medication adherence, and other relevant variables were assessed from 1 week prior to the scheduled quit date through 12 weeks postquit. Participants also received medication reminder prompts (described in the Medication REM section). Those who responded that they did not take their medication as prescribed were asked about their reasons for nonadherence. Participants were compensated up to US $10 per week for the completion of daily diary assessments (up to US $130 total).

Your response is too large. Try shortening some answers.

5-viii) Mode of delivery, features/functionalities/components of the intervention and comparator, and the theoretical framework

Describe mode of delivery, features/functionalities/components of the intervention and comparator, and the theoretical framework [6] used to design them (instructional strategy [1], behaviour change techniques, persuasive features, etc., see e.g., [7, 8] for terminology). This includes an in-depth description of the content (including where it is coming from and who developed it) [1]," whether [and how] it is tailored to individual circumstances and allows users to track their progress and receive feedback" [6]. This also includes a description of communication delivery channels and – if computer-mediated communication is a component – whether communication was synchronous or asynchronous [6]. It also includes information on presentation strategies [1], including page design principles, average amount of text on pages, presence of hyperlinks to other resources, etc. [1].

subitem not at all important

1 ○

2 ○

3 ◉

4 ○

5 ○

essential

Clear selection

Your response is too large. Try shortening some answers.

Does your paper address subitem 5-viii? *

Copy and paste relevant sections from the manuscript (include quotes in quotation marks "like this" to indicate direct quotes from your manuscript), or elaborate on this item by providing additional information not in the ms, or briefly explain why the item is not applicable/relevant for your study

Participants were prompted via the Insight smartphone app [42] to complete daily diary assessments 30 minutes after their self-reported usual wake time. Daily smoking, medication adherence, and other relevant variables were assessed from 1 week prior to the scheduled quit date through 12 weeks postquit. Participants also received medication reminder prompts (described in the Medication REM section). Those who responded that they did not take their medication as prescribed were asked about their reasons for nonadherence. Participants were compensated up to US $10 per week for the completion of daily diary assessments (up to US $130 total).

5-ix) Describe use parameters

Describe use parameters (e.g., intended “doses” and optimal timing for use). Clarify what instructions or recommendations were given to the user, e.g., regarding timing, frequency, heaviness of use, if any, or was the intervention used ad libitum.

subitem not at all important

1 ○

2 ○

3 ◉

4 ○

5 ○

essential

Clear selection

Your response is too large. Try shortening some answers.

Does your paper address subitem 5-ix?

Copy and paste relevant sections from the manuscript (include quotes in quotation marks "like this" to indicate direct quotes from your manuscript), or elaborate on this item by providing additional information not in the ms, or briefly explain why the item is not applicable/relevant for your study

Participants were prompted via the Insight smartphone app [42] to complete daily diary assessments 30 minutes after their self-reported usual wake time. Daily smoking, medication adherence, and other relevant variables were assessed from 1 week prior to the scheduled quit date through 12 weeks postquit. Participants also received medication reminder prompts (described in the Medication REM section). Those who responded that they did not take their medication as prescribed were asked about their reasons for nonadherence. Participants were compensated up to US $10 per week for the completion of daily diary assessments (up to US $130 total).

5-x) Clarify the level of human involvement

Clarify the level of human involvement (care providers or health professionals, also technical assistance) in the e-intervention or as co-intervention (detail number and expertise of professionals involved, if any, as well as “type of assistance offered, the timing and frequency of the support, how it is initiated, and the medium by which the assistance is delivered”. It may be necessary to distinguish between the level of human involvement required for the trial, and the level of human involvement required for a routine application outside of a RCT setting (discuss under item 21 – generalizability).

subitem not at all important

1 ○

2 ○

3 ◉

4 ○

5 ○

essential

Clear selection

Your response is too large. Try shortening some answers.

Does your paper address subitem 5-x?

Copy and paste relevant sections from the manuscript (include quotes in quotation marks "like this" to indicate direct quotes from your manuscript), or elaborate on this item by providing additional information not in the ms, or briefly explain why the item is not applicable/relevant for your study

Participants were initially asked to complete study follow-up assessments in person at the TTRP, but the onset of the COVID-19 pandemic led the university to limit in-person interactions on campus. As a result, between March and May 2020, all assessments were completed remotely via web-based assessments, daily smartphone assessments, and smartphone-based breath sample submissions. After the university reopened, participants were encouraged to attend study visits in person, but remote assessments were accommodated.
Counseling
Approximately 1-week prior to the scheduled quit date, a Tobacco Treatment Specialist (TTS) provided an overview of the tobacco cessation program and assisted participants with developing a quit plan. Participants were instructed to quit smoking at bedtime or 10 PM (whichever occurred first) on the evening before their next weekly counseling session (≈1 week after enrollment). All participants were offered up to 5 additional weekly counseling sessions delivered in-person or by telephone with a TTS. Topics of discussion during the counseling sessions included (1) the impact of tobacco on the health benefits of quitting, (2) stress management strategies, (3) making positive lifestyle changes, (4) developing coping skills, and (5) relapse prevention. The TTS checked in with participants each week about the difficulties and successes they experienced and planned for anticipated future challenges.

Your response is too large. Try shortening some answers.

5-xi) Report any prompts/reminders used

Report any prompts/reminders used: Clarify if there were prompts (letters, emails, phone calls, SMS) to use the application, what triggered them, frequency etc. It may be necessary to distinguish between the level of prompts/reminders required for the trial, and the level of prompts/reminders for a routine application outside of a RCT setting (discuss under item 21 – generalizability).

subitem not at all important

1 ○

2 ○

3 ◉

4 ○

5 ○

essential

Clear selection

Does your paper address subitem 5-xi? *

Copy and paste relevant sections from the manuscript (include quotes in quotation marks "like this" to indicate direct quotes from your manuscript), or elaborate on this item by providing additional information not in the ms, or briefly explain why the item is not applicable/relevant for your study

Participants were prompted via the Insight smartphone app [42] to complete daily diary assessments 30 minutes after their self-reported usual wake time. Daily smoking, medication adherence, and other relevant variables were assessed from 1 week prior to the scheduled quit date through 12 weeks postquit. Participants also received medication reminder prompts (described in the Medication REM section). Those who responded that they did not take their medication as prescribed were asked about their reasons for nonadherence. Participants were compensated up to US $10 per week for the completion of daily diary assessments (up to US $130 total).

Your response is too large. Try shortening some answers.

5-xii) Describe any co-interventions (incl. training/support)

Describe any co-interventions (incl. training/support): Clearly state any interventions that are provided in addition to the targeted eHealth intervention, as ehealth intervention may not be designed as stand-alone intervention. This includes training sessions and support [1]. It may be necessary to distinguish between the level of training required for the trial, and the level of training for a routine application outside of a RCT setting (discuss under item 21 – generalizability.

subitem not at all important

- 1 ○
- 2 ○
- 3 ◉
- 4 ○
- 5 ○

essential

Clear selection

Your response is too large. Try shortening some answers.

Does your paper address subitem 5-xii? *

Copy and paste relevant sections from the manuscript (include quotes in quotation marks "like this" to indicate direct quotes from your manuscript), or elaborate on this item by providing additional information not in the ms, or briefly explain why the item is not applicable/relevant for your study

Counseling
Approximately 1-week prior to the scheduled quit date, a Tobacco Treatment Specialist (TTS) provided an overview of the tobacco cessation program and assisted participants with developing a quit plan. Participants were instructed to quit smoking at bedtime or 10 PM (whichever occurred first) on the evening before their next weekly counseling session (≈1 week after enrollment). All participants were offered up to 5 additional weekly counseling sessions delivered in-person or by telephone with a TTS. Topics of discussion during the counseling sessions included (1) the impact of tobacco on the health benefits of quitting, (2) stress management strategies, (3) making positive lifestyle changes, (4) developing coping skills, and (5) relapse prevention. The TTS checked in with participants each week about the difficulties and successes they experienced and planned for anticipated future challenges.
Varenicline
Participants were evaluated by a study physician, and varenicline was prescribed. Doses were titrated during the first week (prequit) following enrollment (days 1-3: 0.5 mg once daily; days 4-7: 0.5 mg twice daily). Thereafter, participants were instructed to take 0.5 mg varenicline twice daily for 12 additional postquit weeks.
Oral NRT
Participants randomly assigned to VAR + NRT were provided a supply of 2 or 4 mg nicotine lozenges or gum based on their prequit level of smoking (per package instructions) and their preference for gum or lozenges. Participants were instructed to start using gum or lozenges on their quit date and especially when they had the urge to smoke. Participants were initially instructed to use gum or lozenge every 1-2 hours during weeks 1-6 and to gradually reduce to every 2-4 hours during weeks 7-9 and 4-8 hours during weeks 10-12 after the scheduled quit date (per package instructions).

6a) Completely defined pre-specified primary and secondary outcome measures, including how and when they were assessed

Your response is too large. Try shortening some answers.

Does your paper address CONSORT subitem 6a? *

Copy and paste relevant sections from the manuscript (include quotes in quotation marks "like this" to indicate direct quotes from your manuscript), or elaborate on this item by providing additional information not in the ms, or briefly explain why the item is not applicable/relevant for your study

Your response is too large. Try shortening some answers.

Smoking Abstinence
The primary study outcomes were self-reported and biochemically verified 7-day point prevalence abstinence at 4, 8, 12, and 26 weeks after the scheduled quit date. CO was assessed using a portable Vitalograph ecolyzer (Vitalograph Incorporated; for in-clinic assessments) or via the Bedfont iCO Smokerlyzer (Bedfont Scientific Ltd; for remote smartphone-based assessments) with a CO level of ≤6 ppm indicating biochemical confirmation of self-reported abstinence (per current recommendations [35]).
Participants were considered abstinent each day if they did not report smoking during any smartphone-based reminder assessment on that day (see also reasons for non-adherence below), and they reported that they did not smoke during the previous day on the morning daily diary. A variable reflecting the total number of days each participant was abstinent over the post-quit period was created with a possible range of 0-84 days abstinent (ie, the first 12 weeks after the scheduled quit date).
Varenicline Adherence
Participants responded to the following smartphone daily diary question each morning and evening (depending on where they were in the titration schedule): “Have you taken your Chantix pill this [morning/evening]?” A dichotomous variable was created to indicate whether or not the participant had reported using varenicline as prescribed on a given day. For example, if a participant was supposed to take the medication once in the morning and once in the evening, but they reported only taking it once, they were considered nonadherent for that day. The total days of varenicline adherence were calculated with a possible range of 0-91 days (ie, 1 week before the quit date [titration period] through 12 weeks after the scheduled quit date).
NRT Adherence
During each morning daily diary, participants were asked via smartphone assessment, “How many pieces of nicotine gum or lozenges did you use yesterday?” The total pieces of NRT used were reflected as a continuous variable and were later dichotomized to <5 versus ≥5 pieces for each day.
The Medication Adherence Questionnaire (MAQ) [43] is a 4-item participant self-report of adherence to their smoking cessation medications during the previous week. Scores may range from 0 to 4 with higher scores indicating greater medication adherence. The MAQ was administered weekly from the scheduled quit date through 4 weeks after the scheduled quit date and again at the 8- and 12-week postquit follow-ups (7 assessments). Weekly scores were averaged across assessments to create an average adherence score. MAQ scores of 0 were assigned to those who informed study staff that they were dropping out of the study or discontinuing study medication during the weeks after they dropped out. Otherwise, a mean substitution for that week was used in place of missing MAQ scores. MAQ scores were dichotomized based on a median split to reflect higher versus lower adherence within the sample (<3.60 vs ≥3.60).

Your response is too large. Try shortening some answers.

6a-i) Online questionnaires: describe if they were validated for online use and apply CHERRIES items to describe how the questionnaires were designed/deployed

If outcomes were obtained through online questionnaires, describe if they were validated for online use and apply CHERRIES items to describe how the questionnaires were designed/deployed [9].

subitem not at all important

- ○ 1
- ○ 2
- ● 3
- ○ 4
- ○ 5

essential

Clear selection

Your response is too large. Try shortening some answers.

Does your paper address subitem 6a-i?

Copy and paste relevant sections from manuscript text

Smoking Abstinence
The primary study outcomes were self-reported and biochemically verified 7-day point prevalence abstinence at 4, 8, 12, and 26 weeks after the scheduled quit date. CO was assessed using a portable Vitalograph ecolyzer (Vitalograph Incorporated; for in-clinic assessments) or via the Bedfont iCO Smokerlyzer (Bedfont Scientific Ltd; for remote smartphone-based assessments) with a CO level of ≤6 ppm indicating biochemical confirmation of self-reported abstinence (per current recommendations [35]).
Participants were considered abstinent each day if they did not report smoking during any smartphone-based reminder assessment on that day (see also reasons for non-adherence below), and they reported that they did not smoke during the previous day on the morning daily diary. A variable reflecting the total number of days each participant was abstinent over the post-quit period was created with a possible range of 0-84 days abstinent (ie, the first 12 weeks after the scheduled quit date).
Varenicline Adherence
Participants responded to the following smartphone daily diary question each morning and evening (depending on where they were in the titration schedule): “Have you taken your Chantix pill this [morning/evening]?” A dichotomous variable was created to indicate whether or not the participant had reported using varenicline as prescribed on a given day. For example, if a participant was supposed to take the medication once in the morning and once in the evening, but they reported only taking it once, they were considered nonadherent for that day. The total days of varenicline adherence were calculated with a possible range of 0-91 days (ie, 1 week before the quit date [titration period] through 12 weeks after the scheduled quit date).
NRT Adherence
During each morning daily diary, participants were asked via smartphone assessment, “How many pieces of nicotine gum or lozenges did you use yesterday?” The total pieces of NRT used were reflected as a continuous variable and were later dichotomized to <5 versus ≥5 pieces for each day.
The Medication Adherence Questionnaire (MAQ) [43] is a 4-item participant self-report of adherence to their smoking cessation medications during the previous week. Scores may range from 0 to 4 with higher scores indicating greater medication adherence. The MAQ was administered weekly from the scheduled quit date through 4 weeks after the scheduled quit date and again at the 8- and 12-week postquit follow-ups (7 assessments). Weekly scores were averaged across assessments to create an average adherence score. MAQ scores of 0 were assigned to those who informed study staff that they were dropping out of the study or discontinuing study medication during the weeks after they dropped out. Otherwise, a mean substitution for that week was used in place of missing MAQ scores. MAQ scores were dichotomized based on a median split to reflect higher versus lower adherence within the sample (<3.60 vs ≥3.60).

Your response is too large. Try shortening some answers.

6a-ii) Describe whether and how “use” (including intensity of use/dosage) was defined/measured/monitored

Describe whether and how “use” (including intensity of use/dosage) was defined/measured/monitored (logins, logfile analysis, etc.). Use/adoption metrics are important process outcomes that should be reported in any ehealth trial.

subitem not at all important

- 1 ○
- 2 ○
- 3 ◉
- 4 ○
- 5 ○

essential

Clear selection

Your response is too large. Try shortening some answers.

Does your paper address subitem 6a-ii?

Copy and paste relevant sections from manuscript text

Smoking Abstinence
The primary study outcomes were self-reported and biochemically verified 7-day point prevalence abstinence at 4, 8, 12, and 26 weeks after the scheduled quit date. CO was assessed using a portable Vitalograph ecolyzer (Vitalograph Incorporated; for in-clinic assessments) or via the Bedfont iCO Smokerlyzer (Bedfont Scientific Ltd; for remote smartphone-based assessments) with a CO level of ≤6 ppm indicating biochemical confirmation of self-reported abstinence (per current recommendations [35]).
Participants were considered abstinent each day if they did not report smoking during any smartphone-based reminder assessment on that day (see also reasons for non-adherence below), and they reported that they did not smoke during the previous day on the morning daily diary. A variable reflecting the total number of days each participant was abstinent over the post-quit period was created with a possible range of 0-84 days abstinent (ie, the first 12 weeks after the scheduled quit date).
Varenicline Adherence
Participants responded to the following smartphone daily diary question each morning and evening (depending on where they were in the titration schedule): "Have you taken your Chantix pill this [morning/evening]?" A dichotomous variable was created to indicate whether or not the participant had reported using varenicline as prescribed on a given day. For example, if a participant was supposed to take the medication once in the morning and once in the evening, but they reported only taking it once, they were considered nonadherent for that day. The total days of varenicline adherence were calculated with a possible range of 0-91 days (ie, 1 week before the quit date [titration period] through 12 weeks after the scheduled quit date).
NRT Adherence
During each morning daily diary, participants were asked via smartphone assessment, "How many pieces of nicotine gum or lozenges did you use yesterday?" The total pieces of NRT used were reflected as a continuous variable and were later dichotomized to <5 versus ≥5 pieces for each day.
The Medication Adherence Questionnaire (MAQ) [43] is a 4-item participant self-report of adherence to their smoking cessation medications during the previous week. Scores may range from 0 to 4 with higher scores indicating greater medication adherence. The MAQ was administered weekly from the scheduled quit date through 4 weeks after the scheduled quit date and again at the 8- and 12-week postquit follow-ups (7 assessments). Weekly scores were averaged across assessments to create an average adherence score. MAQ scores of 0 were assigned to those who informed study staff that they were dropping out of the study or discontinuing study medication during the weeks after they dropped out. Otherwise, a mean substitution for that week was used in place of missing MAQ scores. MAQ scores were dichotomized based on a median split to reflect higher versus lower adherence within the sample (<3.60 vs ≥3.60).

Your response is too large. Try shortening some answers.

6a-iii) Describe whether, how, and when qualitative feedback from participants was obtained

Describe whether, how, and when qualitative feedback from participants was obtained (e.g., through emails, feedback forms, interviews, focus groups).

subitem not at all important

1 ○

2 ○

3 ◉

4 ○

5 ○

essential

Clear selection

Does your paper address subitem 6a-iii?

Copy and paste relevant sections from manuscript text

During smartphone assessments when participants reported that they had not taken their varenicline or NRT, they were prompted to select the main reason for missing their medication. Participants could choose from the following 7 responses: "I forgot to take it, I was away from home, I experienced side effects, I didn't feel like I needed it, I didn't think it is working, I have decided not to quit smoking," or "other," where they were able to write in their response. Similarly, participants who reported missing their NRT could choose from the following 9 options, "I didn't feel like I needed it, I forgot to take it, I didn't have it with me, I don't think it is working, I ran out, I experienced side effects, I have decided not to quit smoking, I did not like the taste," and "other," which included a

6b) Any changes to trial outcomes after the trial commenced, with reasons

Your response is too large. Try shortening some answers.

Does your paper address CONSORT subitem 6b? *

Copy and paste relevant sections from the manuscript (include quotes in quotation marks "like this" to indicate direct quotes from your manuscript), or elaborate on this item by providing additional information not in the ms, or briefly explain why the item is not applicable/relevant for your study

No - outcomes were not changed

7a) How sample size was determined

NPT: When applicable, details of whether and how the clustering by care provides or centers was addressed

7a-i) Describe whether and how expected attrition was taken into account when calculating the sample size

Describe whether and how expected attrition was taken into account when calculating the sample size.

subitem not at all important

1 ○

2 ○

3 ◉

4 ○

5 ○

essential

Clear selection

Your response is too large. Try shortening some answers.

Does your paper address subitem 7a-i?

Copy and paste relevant sections from manuscript title (include quotes in quotation marks "like this" to indicate direct quotes from your manuscript), or elaborate on this item by providing additional information not in the ms, or briefly explain why the item is not applicable/relevant for your study

N/A pilot study was limited in sample size due to COVID and Chantix recall.

7b) When applicable, explanation of any interim analyses and stopping guidelines

Does your paper address CONSORT subitem 7b? *

Copy and paste relevant sections from the manuscript (include quotes in quotation marks "like this" to indicate direct quotes from your manuscript), or elaborate on this item by providing additional information not in the ms, or briefly explain why the item is not applicable/relevant for your study

We report on data throughout the study period, from pre-quit to 24 weeks post quit.

8a) Method used to generate the random allocation sequence

NPT: When applicable, how care providers were allocated to each trial group

Does your paper address CONSORT subitem 8a? *

Copy and paste relevant sections from the manuscript (include quotes in quotation marks "like this" to indicate direct quotes from your manuscript), or elaborate on this item by providing additional information not in the ms, or briefly explain why the item is not applicable/relevant for your study

Proc plan in SAS version 9.4 was used to create the randomization allocation

Your response is too large. Try shortening some answers.

8b) Type of randomisation; details of any restriction (such as blocking and block size)

Does your paper address CONSORT subitem 8b? *

Copy and paste relevant sections from the manuscript (include quotes in quotation marks "like this" to indicate direct quotes from your manuscript), or elaborate on this item by providing additional information not in the ms, or briefly explain why the item is not applicable/relevant for your study

· Stratified randomization was used to balance the following factors:

o Race – white vs non-white

o Sex – male vs female

o Cigarettes smoked per day – less than 20 vs 20 or more

o Education – GED vs finished HS or less (no GED) vs. more than HS education

· A block size of 12 was used to allocate to the 4 treatment groups in this study within these strata

9) Mechanism used to implement the random allocation sequence (such as sequentially numbered containers), describing any steps taken to conceal the sequence until interventions were assigned

Does your paper address CONSORT subitem 9? *

Copy and paste relevant sections from the manuscript (include quotes in quotation marks "like this" to indicate direct quotes from your manuscript), or elaborate on this item by providing additional information not in the ms, or briefly explain why the item is not applicable/relevant for your study

Randomization is housed inside the REDCap project such that only the statistician knows the allocations or has access to their order… until an allocation has been, well,

Your response is too large. Try shortening some answers.

10) Who generated the random allocation sequence, who enrolled participants, and who assigned participants to interventions

Does your paper address CONSORT subitem 10? *

Copy and paste relevant sections from the manuscript (include quotes in quotation marks "like this" to indicate direct quotes from your manuscript), or elaborate on this item by providing additional information not in the ms, or briefly explain why the item is not applicable/relevant for your study

Research team - biostatistician, research technicians

11a) If done, who was blinded after assignment to interventions (for example, participants, care providers, those assessing outcomes) and how

NPT: Whether or not administering co-interventions were blinded to group assignment

11a-i) Specify who was blinded, and who wasn’t

Specify who was blinded, and who wasn’t. Usually, in web-based trials it is not possible to blind the participants [1, 3] (this should be clearly acknowledged), but it may be possible to blind outcome assessors, those doing data analysis or those administering co-interventions (if any).

subitem not at all important

- 1 ○
- 2 ○
- 3 ◉
- 4 ○
- 5 ○

essential

Your response is too large. Try shortening some answers.

Does your paper address subitem 11a-i? *

Copy and paste relevant sections from the manuscript (include quotes in quotation marks "like this" to indicate direct quotes from your manuscript), or elaborate on this item by providing additional information not in the ms, or briefly explain why the item is not applicable/relevant for your study

N/A the study was unblinded

11a-ii) Discuss e.g., whether participants knew which intervention was the “intervention of interest” and which one was the “comparator”

Informed consent procedures (4a-ii) can create biases and certain expectations - discuss e.g., whether participants knew which intervention was the “intervention of interest” and which one was the “comparator”.

subitem not at all important

- 1 ○
- 2 ○
- 3 ◉
- 4 ○
- 5 ○

essential

Clear selection

Does your paper address subitem 11a-ii?

Copy and paste relevant sections from the manuscript (include quotes in quotation marks "like this" to indicate direct quotes from your manuscript), or elaborate on this item by providing additional information not in the ms, or briefly explain why the item is not applicable/relevant for your study

Participants knew what groups they were assigned to and had access to the protocol IRB

Your response is too large. Try shortening some answers.

11b) If relevant, description of the similarity of interventions
(this item is usually not relevant for ehealth trials as it refers to similarity of a placebo or sham intervention to a active medication/intervention)

Does your paper address CONSORT subitem 11b? *

Copy and paste relevant sections from the manuscript (include quotes in quotation marks "like this" to indicate direct quotes from your manuscript), or elaborate on this item by providing additional information not in the ms, or briefly explain why the item is not applicable/relevant for your study

The intervention is clearly laid out in the methods section.

12a) Statistical methods used to compare groups for primary and secondary outcomes
NPT: When applicable, details of whether and how the clustering by care providers or centers was addressed

Your response is too large. Try shortening some answers.

Does your paper address CONSORT subitem 12a? *

Copy and paste relevant sections from the manuscript (include quotes in quotation marks "like this" to indicate direct quotes from your manuscript), or elaborate on this item by providing additional information not in the ms, or briefly explain why the item is not applicable/relevant for your study

Participants' sociodemographic characteristics were summarized using frequencies with associated percentages (categorical or binary outcomes) or means with SDs (continuous outcomes). Likewise, the percentage of participants who achieved 7-day self-reported and biochemically verified abstinence at follow-up was described by medication type (VAR vs VAR+NRT) and medication reminder group (REM vs NREM). Participants who did not have complete smoking status data at follow-up were considered smoking (ie, self-reported abstinence but did not provide CO breath sample or were missing both self-reported smoking status and CO breath sample). Due to the early discontinuation of the study as a result of the varenicline recall, randomization across groups was uneven, thus, limiting the ability to describe study outcomes across all 4 groups (eg, only 2 participants were randomized to VAR+REM). Thus, descriptive analyses focus on comparisons between the 2 intervention factors (VAR vs VAR+NRT and REM vs NREM).
Participants reported on their tobacco use and medication adherence via smartphone-based daily dairies over the 13-week treatment period (1 week prequit through 12 weeks postquit) with a total of 84 (tobacco use and NRT use) to 91 (varenicline adherence) assessments possible for each outcome. Due to sample size limitations, differences by the treatment factors were described but not compared for statistical significance. Data were aggregated for each individual participant. Daily abstinence and medication adherence were described by medication type (VAR vs VAR+NRT) and reminder group (REM vs NREM).

Your response is too large. Try shortening some answers.

12a-i) Imputation techniques to deal with attrition / missing values

Imputation techniques to deal with attrition / missing values: Not all participants will use the intervention/comparator as intended and attrition is typically high in ehealth trials. Specify how participants who did not use the application or dropped out from the trial were treated in the statistical analysis (a complete case analysis is strongly discouraged, and simple imputation techniques such as LOCF may also be problematic [4]).

subitem not at all important

1 ○

2 ○

3 ◉

4 ○

5 ○

essential

Clear selection

Does your paper address subitem 12a-i? *

Copy and paste relevant sections from the manuscript (include quotes in quotation marks "like this" to indicate direct quotes from your manuscript), or elaborate on this item by providing additional information not in the ms, or briefly explain why the item is not applicable/relevant for your study

Did not use imputation in this as we

12b) Methods for additional analyses, such as subgroup analyses and adjusted analyses

Your response is too large. Try shortening some answers.

Does your paper address CONSORT subitem 12b? *

Copy and paste relevant sections from the manuscript (include quotes in quotation marks "like this" to indicate direct quotes from your manuscript), or elaborate on this item by providing additional information not in the ms, or briefly explain why the item is not applicable/relevant for your study

Analyses are reported by intervention group

X26) REB/IRB Approval and Ethical Considerations [recommended as subheading under "Methods"] (not a CONSORT item)

X26-i) Comment on ethics committee approval

subitem not at all important

1 ○

2 ○

3 ◉

4 ○

5 ○

essential

Clear selection

Your response is too large. Try shortening some answers.

Does your paper address subitem X26-i?

Copy and paste relevant sections from the manuscript (include quotes in quotation marks "like this" to indicate direct quotes from your manuscript), or elaborate on this item by providing additional information not in the ms, or briefly explain why the item is not applicable/relevant for your study

This study was approved by the Institutional Review Board of the University of Oklahoma Health Sciences Center (protocol 10184). Informed consent was obtained from all participants. Privacy and confidentiality were maintained by assigning an identification number in place of names in all secured electronic and print data files. Participants were able to earn up to US $250 in total compensation (gift cards) for assessment completions over the entire study period.

x26-ii) Outline informed consent procedures

Outline informed consent procedures e.g., if consent was obtained offline or online (how? Checkbox, etc.?), and what information was provided (see 4a-ii). See [6] for some items to be included in informed consent documents.

subitem not at all important

- 1 ○
- 2 ○
- 3 ◉
- 4 ○
- 5 ○

essential

Clear selection

Your response is too large. Try shortening some answers.

Does your paper address subitem X26-ii?

Copy and paste relevant sections from the manuscript (include quotes in quotation marks "like this" to indicate direct quotes from your manuscript), or elaborate on this item by providing additional information not in the ms, or briefly explain why the item is not applicable/relevant for your study

This study was approved by the Institutional Review Board of the University of Oklahoma Health Sciences Center (protocol 10184). Informed consent was obtained from all participants. Privacy and confidentiality were maintained by assigning an identification number in place of names in all secured electronic and print data files. Participants were able to earn up to US $250 in total compensation (gift cards) for assessment completions over the entire study period.

X26-iii) Safety and security procedures

Safety and security procedures, incl. privacy considerations, and any steps taken to reduce the likelihood or detection of harm (e.g., education and training, availability of a hotline)

subitem not at all important

- 1 ○
- 2 ○
- 3 ◉
- 4 ○
- 5 ○

essential

Clear selection

Your response is too large. Try shortening some answers.

Does your paper address subitem X26-iii?

Copy and paste relevant sections from the manuscript (include quotes in quotation marks "like this" to indicate direct quotes from your manuscript), or elaborate on this item by providing additional information not in the ms, or briefly explain why the item is not applicable/relevant for your study

Informed consent was obtained from all participants. Privacy and confidentiality were maintained by assigning an identification number in place of names in all secured electronic and print data files.

RESULTS

13a) For each group, the numbers of participants who were randomly assigned, received intended treatment, and were analysed for the primary outcome

NPT: The number of care providers or centers performing the intervention in each group and the number of patients treated by each care provider in each center

Does your paper address CONSORT subitem 13a? *

Copy and paste relevant sections from the manuscript (include quotes in quotation marks "like this" to indicate direct quotes from your manuscript), or elaborate on this item by providing additional information not in the ms, or briefly explain why the item is not applicable/relevant for your study

This is shown in Table 1 with the breakdown of how many participants are in each group

13b) For each group, losses and exclusions after randomisation, together with reasons

Your response is too large. Try shortening some answers.

Does your paper address CONSORT subitem 13b? (NOTE: Preferably, this is shown in a CONSORT flow diagram) *

Copy and paste relevant sections from the manuscript (include quotes in quotation marks "like this" to indicate direct quotes from your manuscript), or elaborate on this item by providing additional information not in the ms, or briefly explain why the item is not applicable/relevant for your study

This is not described in the paper, there was no notable attrition

13b-i) Attrition diagram

Strongly recommended: An attrition diagram (e.g., proportion of participants still logging in or using the intervention/comparator in each group plotted over time, similar to a survival curve) or other figures or tables demonstrating usage/dose/engagement.

subitem not at all important

- 1 ○
- 2 ○
- 3 ◉
- 4 ○
- 5 ○

essential

Clear selection

Does your paper address subitem 13b-i?

Copy and paste relevant sections from the manuscript or cite the figure number if applicable (include quotes in quotation marks "like this" to indicate direct quotes from your manuscript), or elaborate on this item by providing additional information not in the ms, or briefly explain why the item is not applicable/relevant for your study

No formal dropouts reported

Your response is too large. Try shortening some answers.

14a) Dates defining the periods of recruitment and follow-up

Does your paper address CONSORT subitem 14a? *

Copy and paste relevant sections from the manuscript (include quotes in quotation marks "like this" to indicate direct quotes from your manuscript), or elaborate on this item by providing additional information not in the ms, or briefly explain why the item is not applicable/relevant for your study

We have included this by weeks since quit date

14a-i) Indicate if critical "secular events" fell into the study period

Indicate if critical "secular events" fell into the study period, e.g., significant changes in Internet resources available or "changes in computer hardware or Internet delivery resources"

subitem not at all important

- 1 ○
- 2 ○
- 3 ◉
- 4 ○
- 5 ○

essential

Clear selection

Your response is too large. Try shortening some answers.

Does your paper address subitem 14a-i?

Copy and paste relevant sections from the manuscript (include quotes in quotation marks "like this" to indicate direct quotes from your manuscript), or elaborate on this item by providing additional information not in the ms, or briefly explain why the item is not applicable/relevant for your study

Your answer

14b) Why the trial ended or was stopped (early)

Does your paper address CONSORT subitem 14b? *

Copy and paste relevant sections from the manuscript (include quotes in quotation marks "like this" to indicate direct quotes from your manuscript), or elaborate on this item by providing additional information not in the ms, or briefly explain why the item is not applicable/relevant for your study

Due to the early discontinuation of the study as a result of the varenicline recall, randomization across groups was uneven, thus, limiting the ability to describe study outcomes across all 4 groups (eg, only 2 participants were randomized to VAR+REM). Thus, descriptive analyses focus on comparisons between the 2 intervention factors (VAR vs VAR+NRT and REM vs NREM).

15) A table showing baseline demographic and clinical characteristics for each group

NPT: When applicable, a description of care providers (case volume, qualification, expertise, etc.) and centers (volume) in each group

Your response is too large. Try shortening some answers.

Does your paper address CONSORT subitem 15? *

Copy and paste relevant sections from the manuscript (include quotes in quotation marks "like this" to indicate direct quotes from your manuscript), or elaborate on this item by providing additional information not in the ms, or briefly explain why the item is not applicable/relevant for your study

Table 2 highlights the demographics

15-i) Report demographics associated with digital divide issues

In ehealth trials it is particularly important to report demographics associated with digital divide issues, such as age, education, gender, social-economic status, computer/Internet/ehealth literacy of the participants, if known.

subitem not at all important

1 ○

2 ○

3 ●

4 ○

5 ○

essential

Clear selection

Does your paper address subitem 15-i? *

Copy and paste relevant sections from the manuscript (include quotes in quotation marks "like this" to indicate direct quotes from your manuscript), or elaborate on this item by providing additional information not in the ms, or briefly explain why the item is not applicable/relevant for your study

We do not report this in this paper

Your response is too large. Try shortening some answers.

16) For each group, number of participants (denominator) included in each analysis and whether the analysis was by original assigned groups

16-i) Report multiple “denominators” and provide definitions

Report multiple “denominators” and provide definitions: Report N’s (and effect sizes) “across a range of study participation [and use] thresholds” [1], e.g., N exposed, N consented, N used more than x times, N used more than y weeks, N participants “used” the intervention/comparator at specific pre-defined time points of interest (in absolute and relative numbers per group). Always clearly define “use” of the intervention.

subitem not at all important

1 ○

2 ○

3 ◉

4 ○

5 ○

essential

Clear selection

Does your paper address subitem 16-i? *

Copy and paste relevant sections from the manuscript (include quotes in quotation marks "like this" to indicate direct quotes from your manuscript), or elaborate on this item by providing additional information not in the ms, or briefly explain why the item is not applicable/relevant for your study

N's are reported for all analysis

Your response is too large. Try shortening some answers.

16-ii) Primary analysis should be intent-to-treat

Primary analysis should be intent-to-treat, secondary analyses could include comparing only “users”, with the appropriate caveats that this is no longer a randomized sample (see 18-i).

subitem not at all important

1 ○

2 ○

3 ◉

4 ○

5 ○

essential

Clear selection

Does your paper address subitem 16-ii?

Copy and paste relevant sections from the manuscript (include quotes in quotation marks "like this" to indicate direct quotes from your manuscript), or elaborate on this item by providing additional information not in the ms, or briefly explain why the item is not applicable/relevant for your study

Your answer

17a) For each primary and secondary outcome, results for each group, and the estimated effect size and its precision (such as 95% confidence interval)

Your response is too large. Try shortening some answers.

Does your paper address CONSORT subitem 17a? *

Copy and paste relevant sections from the manuscript (include quotes in quotation marks "like this" to indicate direct quotes from your manuscript), or elaborate on this item by providing additional information not in the ms, or briefly explain why the item is not applicable/relevant for your study

Descriptive data is shown, 95 CI are n/a

17a-i) Presentation of process outcomes such as metrics of use and intensity of use

In addition to primary/secondary (clinical) outcomes, the presentation of process outcomes such as metrics of use and intensity of use (dose, exposure) and their operational definitions is critical. This does not only refer to metrics of attrition (13-b) (often a binary variable), but also to more continuous exposure metrics such as "average session length". These must be accompanied by a technical description how a metric like a "session" is defined (e.g., timeout after idle time) [1] (report under item 6a).

subitem not at all important

- 1 ○
- 2 ○
- 3 ◉
- 4 ○
- 5 ○

essential

Clear selection

Your response is too large. Try shortening some answers.

Does your paper address subitem 17a-i?

Copy and paste relevant sections from the manuscript (include quotes in quotation marks "like this" to indicate direct quotes from your manuscript), or elaborate on this item by providing additional information not in the ms, or briefly explain why the item is not applicable/relevant for your study

We do not describe these as it isn't the aim of the paper and this is a pilot test

17b) For binary outcomes, presentation of both absolute and relative effect sizes is recommended

Does your paper address CONSORT subitem 17b? *

Copy and paste relevant sections from the manuscript (include quotes in quotation marks "like this" to indicate direct quotes from your manuscript), or elaborate on this item by providing additional information not in the ms, or briefly explain why the item is not applicable/relevant for your study

We do not describe these as it isn't the aim of the paper and this is a pilot test

18) Results of any other analyses performed, including subgroup analyses and adjusted analyses, distinguishing pre-specified from exploratory

Does your paper address CONSORT subitem 18? *

Copy and paste relevant sections from the manuscript (include quotes in quotation marks "like this" to indicate direct quotes from your manuscript), or elaborate on this item by providing additional information not in the ms, or briefly explain why the item is not applicable/relevant for your study

We do not describe these as it isn't the aim of the paper and this is a pilot test

Your response is too large. Try shortening some answers.

18-i) Subgroup analysis of comparing only users

A subgroup analysis of comparing only users is not uncommon in ehealth trials, but if done, it must be stressed that this is a self-selected sample and no longer an unbiased sample from a randomized trial (see 16-iii).

subitem not at all important

1 ○

2 ○

3 ◉

4 ○

5 ○

essential

Clear selection

Does your paper address subitem 18-i?

Copy and paste relevant sections from the manuscript (include quotes in quotation marks "like this" to indicate direct quotes from your manuscript), or elaborate on this item by providing additional information not in the ms, or briefly explain why the item is not applicable/relevant for your study

we did not do this type of analysis

19) All important harms or unintended effects in each group

(for specific guidance see CONSORT for harms)

Your response is too large. Try shortening some answers.

Does your paper address CONSORT subitem 19? *

Copy and paste relevant sections from the manuscript (include quotes in quotation marks "like this" to indicate direct quotes from your manuscript), or elaborate on this item by providing additional information not in the ms, or briefly explain why the item is not applicable/relevant for your study

e report common side effects, Reasons for Nonadherence
Of the specified reasons for missing varenicline doses (n=276 instances throughout the entire study period), the most frequently reported reasons were side effects (27.1%) and forgetting (14.0%). Of the participants who reported varenicline side effects, 77.8% reported
that side effects were mild or very mild, with participants mostly commonly reporting sleep
problems (67.0%) or nausea (29. 3%). Likewise, of the participants who indicated that they
did not use any NRT on a given day (702 instances across all participants throughout the
study period), the most common reasons were “I did not feel like I needed it” (30.81%) and “I
did not have any with me” (14.2%).

19-i) Include privacy breaches, technical problems

Include privacy breaches, technical problems. This does not only include physical “harm” to participants, but also incidents such as perceived or real privacy breaches [1], technical problems, and other unexpected/unintended incidents. “Unintended effects” also includes unintended positive effects [2].

subitem not at all important

1 ○

2 ○

3 ◉

4 ○

5 ○

essential

Your response is too large. Try shortening some answers.

Does your paper address subitem 19-i?

Copy and paste relevant sections from the manuscript (include quotes in quotation marks "like this" to indicate direct quotes from your manuscript), or elaborate on this item by providing additional information not in the ms, or briefly explain why the item is not applicable/relevant for your study

Your answer

19-ii) Include qualitative feedback from participants or observations from staff/researchers

Include qualitative feedback from participants or observations from staff/researchers, if available, on strengths and shortcomings of the application, especially if they point to unintended/unexpected effects or uses. This includes (if available) reasons for why people did or did not use the application as intended by the developers.

subitem not at all important

1 ○

2 ○

3 ○

4 ○

5 ○

essential

Does your paper address subitem 19-ii?

Copy and paste relevant sections from the manuscript (include quotes in quotation marks "like this" to indicate direct quotes from your manuscript), or elaborate on this item by providing additional information not in the ms, or briefly explain why the item is not applicable/relevant for your study

Your answer

Your response is too large. Try shortening some answers.

DISCUSSION

22) Interpretation consistent with results, balancing benefits and harms, and considering other relevant evidence

NPT: In addition, take into account the choice of the comparator, lack of or partial blinding, and unequal expertise of care providers or centers in each group

22-i) Restate study questions and summarize the answers suggested by the data, starting with primary outcomes and process outcomes (use)

Restate study questions and summarize the answers suggested by the data, starting with primary outcomes and process outcomes (use).

subitem not at all important

1 ○

2 ○

3 ◉

4 ○

5 ○

essential

Clear selection

Your response is too large. Try shortening some answers.

Does your paper address subitem 22-i? *

Copy and paste relevant sections from the manuscript (include quotes in quotation marks "like this" to indicate direct quotes from your manuscript), or elaborate on this item by providing additional information not in the ms, or briefly explain why the item is not applicable/relevant for your study

Conclusions: Preliminary findings indicated that smoking cessation interventions may benefit from incorporating medication reminders and combining varenicline with oral NRT, though combining medications may be associated with poorer adherence. Further study is warranted.

22-ii) Highlight unanswered new questions, suggest future research

Highlight unanswered new questions, suggest future research.

subitem not at all important

- 1 ○
- 2 ○
- 3 ◉
- 4 ○
- 5 ○

essential

Clear selection

Your response is too large. Try shortening some answers.

Does your paper address subitem 22-ii?

Copy and paste relevant sections from the manuscript (include quotes in quotation marks "like this" to indicate direct quotes from your manuscript), or elaborate on this item by providing additional information not in the ms, or briefly explain why the item is not applicable/relevant for your study

Conclusions: Preliminary findings indicated that smoking cessation interventions may benefit from incorporating medication reminders and combining varenicline with oral NRT, though combining medications may be associated with poorer adherence. Further study is warranted.

20) Trial limitations, addressing sources of potential bias, imprecision, and, if relevant, multiplicity of analyses

20-i) Typical limitations in ehealth trials

Typical limitations in ehealth trials: Participants in ehealth trials are rarely blinded. Ehealth trials often look at a multiplicity of outcomes, increasing risk for a Type I error. Discuss biases due to non-use of the intervention/usability issues, biases through informed consent procedures, unexpected events.

subitem not at all important

1 ○

2 ○

3 ◉

4 ○

5 ○

essential

Clear selection

Your response is too large. Try shortening some answers.

Does your paper address subitem 20-i? *

Copy and paste relevant sections from the manuscript (include quotes in quotation marks "like this" to indicate direct quotes from your manuscript), or elaborate on this item by providing additional information not in the ms, or briefly explain why the item is not applicable/relevant for your study

key limitation of this study was the small sample size. Due to the recall of varenicline in 2021, the study was discontinued before reaching the planned enrollment of 100 participants. Study analyses were not sufficiently powered to evaluate statistically significant differences between groups or support multilevel analyses. Nevertheless, descriptive analyses and initial study findings provide a starting point for future investigation

21) Generalisability (external validity, applicability) of the trial findings

NPT: External validity of the trial findings according to the intervention, comparators, patients, and care providers or centers involved in the trial

21-i) Generalizability to other populations

Generalizability to other populations: In particular, discuss generalizability to a general Internet population, outside of a RCT setting, and general patient population, including applicability of the study results for other organizations

subitem not at all important

1 ○

2 ○

3 ◉

4 ○

5 ○

essential

Clear selection

Your response is too large. Try shortening some answers.

Does your paper address subitem 21-i?

Copy and paste relevant sections from the manuscript (include quotes in quotation marks "like this" to indicate direct quotes from your manuscript), or elaborate on this item by providing additional information not in the ms, or briefly explain why the item is not applicable/relevant for your study

A full-scale randomized trial will be required before conclusions can be drawn about the efficacy of a combination of varenicline and oral NRT for smoking cessation. If combined varenicline and oral NRT are found to improve cessation rates over varenicline alone, adults who smoke will have a new evidence-based treatment option available to them as they undertake the difficult process of quitting smoking. However, medication regimens that include multiple medications and dosing multiple times per day may pose challenges to adherence [28,50]. Smartphone-based medication adherence interventions may be expanded to include multiple components in addition to reminders, such as education, dosing instructions, and novel smartphone-based medication refill requests [52]. Scheduled smartphone-based medication reminders have the potential to improve both adherence and smoking cessation outcomes

21-ii) Discuss if there were elements in the RCT that would be different in a routine application setting

Discuss if there were elements in the RCT that would be different in a routine application setting (e.g., prompts/reminders, more human involvement, training sessions or other co-interventions) and what impact the omission of these elements could have on use, adoption, or outcomes if the intervention is applied outside of a RCT setting.

subitem not at all important

1 ○

2 ○

3 ◉

4 ○

5 ○

essential

Clear selection

Your response is too large. Try shortening some answers.

Does your paper address subitem 21-ii?

Copy and paste relevant sections from the manuscript (include quotes in quotation marks "like this" to indicate direct quotes from your manuscript), or elaborate on this item by providing additional information not in the ms, or briefly explain why the item is not applicable/relevant for your study

Your answer

OTHER INFORMATION

23) Registration number and name of trial registry

Does your paper address CONSORT subitem 23? *

Copy and paste relevant sections from the manuscript (include quotes in quotation marks "like this" to indicate direct quotes from your manuscript), or elaborate on this item by providing additional information not in the ms, or briefly explain why the item is not applicable/relevant for your study

Trial Registration: ClinicalTrials.gov NCT0372296

24) Where the full trial protocol can be accessed, if available

Does your paper address CONSORT subitem 24? *

Cite a Multimedia Appendix, other reference, or copy and paste relevant sections from the manuscript (include quotes in quotation marks "like this" to indicate direct quotes from your manuscript), or elaborate on this item by providing additional information not in the ms, or briefly explain why the item is not applicable/relevant for your study

Trial Registration: ClinicalTrials.gov NCT0372296

Your response is too large. Try shortening some answers.

25) Sources of funding and other support (such as supply of drugs), role of funders

Does your paper address CONSORT subitem 25? *

Copy and paste relevant sections from the manuscript (include quotes in quotation marks "like this" to indicate direct quotes from your manuscript), or elaborate on this item by providing additional information not in the ms, or briefly explain why the item is not applicable/relevant for your study

This study was primarily supported by the Oklahoma Tobacco Settlement Endowment Trust (contract R21-02). Varenicline was provided by Pfizer (WI246498). Additional support was provided by National Cancer Institute Cancer Center Support Grant P30CA225520 awarded to the Stephenson Cancer Center/Mobile Health Shared Resource and The National Institutes of Health/National Institute on Drug Abuse grant K99DA046564/R00DA046564 (to EH). This trial is registered at ClinicalTrials.gov (NCT03722966).udy was primarily supported by the Oklahoma Tobacco Settlement Endowment Trust (contract R21-02). Varenicline was provided by Pfizer (WI246498). Additional support was provided by National Cancer Institute Cancer Center Support Grant P30CA225520 awarded to the Stephenson Cancer Center/Mobile Health Shared Resource and The National Institutes of Health/National Institute on Drug Abuse grant K99DA046564/R00DA046564 (to EH). This trial is registered at ClinicalTrials.gov (NCT03722966)

X27) Conflicts of Interest (not a CONSORT item)

Your response is too large. Try shortening some answers.

X27-i) State the relation of the study team towards the system being evaluated

In addition to the usual declaration of interests (financial or otherwise), also state the relation of the study team towards the system being evaluated, i.e., state if the authors/evaluators are distinct from or identical with the developers/sponsors of the intervention.

subitem not at all important

1 ○

2 ○

3 ◉

4 ○

5 ○

essential

Clear selection

Does your paper address subitem X27-i?

Copy and paste relevant sections from the manuscript (include quotes in quotation marks "like this" to indicate direct quotes from your manuscript), or elaborate on this item by providing additional information not in the ms, or briefly explain why the item is not applicable/relevant for your study

MSB and DEK are inventors of the Insight mHealth Platform, which was used to develop the study app. They receive royalties related to its use. However, they did not receive royalties in this case.

About the CONSORT EHEALTH checklist

Your response is too large. Try shortening some answers.

As a result of using this checklist, did you make changes in your manuscript? *

- ○ yes, major changes
- ◉ yes, minor changes
- ○ no

What were the most important changes you made as a result of using this checklist?

adding blinding classification

How much time did you spend on going through the checklist INCLUDING making changes in your manuscript *

many hours - more than a days work

As a result of using this checklist, do you think your manuscript has improved? *

- ○ yes
- ◉ no
- ○ Other:

Your response is too large. Try shortening some answers.

Would you like to become involved in the CONSORT EHEALTH group?

This would involve for example becoming involved in participating in a workshop and writing an "Explanation and Elaboration" document

- ○ yes
- ◉ no
- ○ Other:

Clear selection

Any other comments or questions on CONSORT EHEALTH

Your answer

STOP - Save this form as PDF before you click submit

To generate a record that you filled in this form, we recommend to generate a PDF of this page (on a Mac, simply select "print" and then select "print as PDF") before you submit it.

When you submit your (revised) paper to JMIR, please upload the PDF as supplementary file.

Don't worry if some text in the textboxes is cut off, as we still have the complete information in our database. Thank you!

Final step: Click submit !

Click submit so we have your answers in our database!

Submit Clear form

Never submit passwords through Google Forms.

This content is neither created nor endorsed by Google. Report Abuse - Terms of Service - Privacy Policy

Your response is too large. Try shortening some answers.

Your response is too large. Try shortening some answers.
